# On De Giorgi’s conjecture of nonlocal approximations for free-discontinuity problems: The symmetric gradient case

**DOI:** 10.1007/s00526-026-03338-w

**Published:** 2026-04-13

**Authors:** S. Almi, E. Davoli, A. Kubin, E. Tasso

**Affiliations:** 1 Dipartimento di Matematica e Applicazioni R. Caccioppoli, Universitá di Napoli Federico II, via Cintia, Monte S. Angelo, 80126 Naples, Italy; 2 Institute of Analysis and Scientific Computing, Wiedner-Hauptstrasse 8-10, 1040 Vienna, Austria

**Keywords:** 49Q20, 49J45, 26B30

## Abstract

We prove that E. De Giorgi’s conjecture for the nonlocal approximation of free-discontinuity problems extends to the case of functionals defined in terms of the symmetric gradient of the admissible field. After introducing a suitable class of continuous finite-difference approximants, we show the compactness of deformations with equibounded energies, as well as their Gamma-convergence. The compactness analysis is a crucial hurdle, which we overcome by generalizing a Fréchet-Kolmogorov approach previously introduced by two of the authors. A second essential difficulty is the identification of the limiting space of admissible deformations, since a control on the directional variations is, a priori, only available in average. A limiting representation in GSBD is eventually established via a novel characterization of this space.

## Introduction

Free-discontinuity problems and their approximation by means of Sobolev formulations, discrete descriptions, or nonlocal functionals are a thriving research area owing to their broad scope of applications, ranging from image reconstruction, to the modeling of failure phenomena in continuum mechanics. A milestone in this direction was a conjecture formulated by E. De Giorgi and proven by M. Gobbino [31], dealing with the approximation via $\Gamma$-convergence of the Mumford-Shah functional by a sequence of nonlocal counterparts. Such analysis has then paved the way for further generalizations in [22, 32]. All the aforementioned contributions deal with free-discontinuity problems involving the full distributional gradient of the admissible maps. A question that, to the authors’ knowledge, was so far left open, was whether similar nonlocal approximation techniques would also prove successful for the study of free-boundary problems involving more general differential operators.

In this paper we initiate the study of *continuous finite-difference approximations* of linearized Griffith functionals. Aside from purely mathematical interest, such inquiries are deeply rooted in the recent research lines which are developing in image processing and data science. Motivated by applications in Magnetic Resonance Imaging (MRI) or Positron Emission Tomography (PET), regularizers involving more general differential operators than the gradient have been studied, e.g., in the settings of regularization graphs [9], for higher-order Total Variation operators [10, 12], as well as in structural Total Variation approaches [33] (see also [13] for a review on data-driven approaches in image regularization). Concerning the data-science applicative interest, starting from the variational analysis in [30], a novel research direction has open up for variational studies on point clouds (see, e.g., [6, 15, 16, 24]), as well as related machine-learning applications [14]. The corresponding mathematical study of generalized graph-based differential constraints different from curl $A=0$ is, to the authors’ knowledge, a thriving, mostly unexplored, subject (see, e.g., [34] and the references therein).

The case study we tackle here is a symmetrized counterpart of the nonlocal energies considered in [31, 32]. In order to describe our findings, we first recall the results [31], where the sequence of functionals

1.1 $$\begin{aligned} \mathscr {F}_{\varepsilon }(u,\Omega ) :=\frac{1}{\varepsilon ^{n+1}}\int _{\Omega \times \Omega } \arctan \left( \frac{( u( x' )-u( x ))^2}{\varepsilon } \right) e^{-|\frac{ x' - x }{\varepsilon }|^2} \text {d}x' \text {d}x, \quad u \in L^1(\Omega ) \end{aligned}$$

is shown to $\Gamma$-converge, as $\varepsilon$ tends to zero, to the Mumford-Shah energy

1.2 $$\begin{aligned} \sqrt{\pi }\mathcal{M}\mathcal{S}_{1/ \sqrt{\pi }}(u,\Omega ) := \int _{\Omega } |\nabla u(x)|^2 \text {d}x + \sqrt{\pi }\mathcal {H}^{n-1}(J_u), \quad u \in \text {GSBV}(\Omega ). \end{aligned}$$

In (1.2), the acronym $\textrm{GSBV}$ stands for the space of functions with generalised special bounded variation (roughly speaking, maps whose truncations are functions of bounded variations with distributional gradients exhibiting null Cantor part), cf. [4, Section 4.5]. Note that the multiplicative constants in (1.2) depend only on the choice of the integrand $\arctan (x)$ and on the weight function $e^{-|x|^2}$.

It is thus natural, in a first stage, to ask whether the functional

1.3 $$\begin{aligned} \mathcal {F}_{\varepsilon }(u,\Omega ) :=\frac{1}{\varepsilon ^{n+1}}\int _{\Omega \times \Omega } \arctan \left( \frac{( (u( x' )-u( x )) \cdot (x' - x) )^2}{\varepsilon ^3} \right) e^{-|\frac{ x' - x }{\varepsilon }|^2} \text {d}x' \text {d}x \end{aligned}$$

defined for measurable functions $u \in L^{0} (\Omega ; \mathbb {R}^{n})$ provides, analogously, a nonlocal approximation, in the sense of $\Gamma$-convergence, of linearized free discontinuity problems of the form

$$\begin{aligned} \mathcal {F}(u,\Omega ) :=\int _{\Omega } \varphi (e(u) ) \, \text {d}x +C\mathcal {H}^{n-1}(J_u), \quad u \in \text {GSBD}(\Omega ) \end{aligned}$$

for suitable choices of the density $\varphi$ and of the constant $C>0$. In the expression above, $\text {GSBD}(\Omega )$ denotes the space of functions with generalized special bounded deformation (see [26]), and *e*(*u*) the absolutely continuous part of the symmetric distributional gradient $\mathcal {E}(u):=\tfrac{Du+Du^{\top }}{2}$.

The structure of (1.3) aligns with the principles of linearized theories in continuum mechanics, where only the symmetric part of the gradient contributes to the deformation energy. This consideration naturally motivates the assumption that only the component of $u(x') - u(x)$ projected along the direction $x'-x$ should provide energy contributions. By setting $\varepsilon \xi :=x' -x$, when the differences $(u(x + \varepsilon \xi ) - u(x)) \cdot \xi$ are relatively small, the functionals in (1.3) behave like pure bulk energies. For large values of $(u(x + \varepsilon \xi ) - u(x)) \cdot \xi$, instead, the energies in (1.3) saturate, effectively detecting and penalizing the size of the discontinuities of *u*.

An important remark concerns the fact that the energies in (1.1) only approximate the Mumford-Shah functional under suitable additional assumptions on the geometry of $\Omega$, such as Lipschitz regularity (see [31, Remark 7.1]). Such requirements may not be fully consistent when handling free-boundary problems where discontinuities are already present within the material. Consider, for example, the case of a crack-initiated domain, where the presence of an $(n-1)$-dimensional set $\Gamma \subset \Omega$, representing an initial crack makes the set $\Omega \setminus \Gamma$ not Lipschitz.

This possible lack of regularity of $\Omega$ calls for a more refined approach, which we address by modifying the energies in (1.3) as follows. First, for every $\xi \in \mathbb {S}^{n-1}$ and every Borel set $E \subset \Omega$ we introduce the functionals $F_{\varepsilon ,\xi }(u,E)$, defined as

$$\begin{aligned} F_{\varepsilon ,\xi }(u,E):=\frac{1}{\varepsilon } \int _{E \cap (E-\varepsilon \xi )} \arctan \left( \frac{((u(x+\varepsilon \xi )-u(x))\cdot \xi )^2}{\varepsilon } \right) \text {d}x,\quad u \in L^0(\Omega ;\mathbb {R}^n). \end{aligned}$$

The specific structure of (1.3) allows one to make use of Fubini’s theorem and rewrite it as an average of $F_{\varepsilon ,\xi }$ with respect to all possible directions $\xi$. Specifically, we have

$$\begin{aligned} \mathcal {F}_{\varepsilon }(u,E) =\int _{\frac{E-E}{\varepsilon }} F_{\varepsilon ,\xi }(u,E) e^{-|\xi |^2} \text {d}\xi , \end{aligned}$$

where $E-E:=\{y \in \mathbb {R}^n: y=x'-x, \text { for some } x,x' \in E\}$. The relevant functionals for our analysis, denoted by $\mathcal {F}^p_\varepsilon (u,\Omega )$, depend on a further degree of freedom, encoded by a parameter $p \in [1,\infty )$, and take the form

1.4 $$\begin{aligned} \mathcal {F}^p_\varepsilon (u,\Omega ):= \sup _{\mathscr {B}} \sum _{B \in \mathscr {B}} \bigg (\int _{\frac{\Omega -\Omega }{\varepsilon }} F_{\varepsilon ,\xi }(u,B)^p e^{-|\xi |^2} \text {d}\xi \bigg )^{\frac{1}{p}},\quad u \in L^0(\Omega ;\mathbb {R}^n). \end{aligned}$$

In the expression above, $\mathscr {B}$ represents the class of all finite families of pairwise disjoint open balls contained in $\Omega$. This supremum procedure over $\mathscr {B}$ allows the functional $\mathcal {F}^{p}_\varepsilon$ to bypass irregularities within the domain $\Omega$. In fact, by choosing $p=1$, the strict superadditivity of the set function $F_{\varepsilon ,\xi }(u,\cdot )$ leads to the strict inequality $\mathcal {F}^1_{\varepsilon } < \mathcal {F}_{\varepsilon }$.

For $p>1$, the presence of the $L^p$-norm in the definition (1.4) should be viewed as a *regularizing effect*.

The major difficulty we face in the analysis of (1.4) is the lack of a clear compactness strategy. The results of [3, 19, 20] cannot be applied, as the sequence $\mathcal {F}^{p}_{\varepsilon }$ is defined over the space of measurable maps, so that neither a differential structure nor integrability assumptions are given a priori. Thus, our main result is the following compactness and closure theorem for sequences $u_{\varepsilon }$ with equibounded energy $\mathcal {F}^{p}_{\varepsilon }$, for some $p \in [1, +\infty )$.

### Theorem 1.1

(Closure and compactness) Let $\Omega \subset \mathbb {R}^n$ be open, let $p\ge 1$, and let $\{u_{\varepsilon }\}_{\varepsilon >0} \subset L^0(\Omega ;\mathbb {R}^n)$ be such that

1.5 $$\begin{aligned} \sup _{\varepsilon >0}\mathcal {F}_{\varepsilon }^p(u_{\varepsilon },\Omega ) < \infty . \end{aligned}$$

Then, there exists a subsequence $\varepsilon _k \rightarrow 0$ as $k \rightarrow \infty$ such that the set

1.6 $$\begin{aligned} A := \{x \in \Omega : |u_{\varepsilon _k}(x)|\rightarrow \infty \,\, \text {as} \,\, k\rightarrow \infty \} \end{aligned}$$

has finite perimeter, and $u_{\varepsilon _k}\rightarrow u$ pointwise almost everywhere in $\Omega \setminus A$ for some measurable function $u :\Omega \setminus A \rightarrow \mathbb {R}^n$. In addition, for almost every $c \in \mathbb {R}$, the extension of *u* to the whole of $\Omega$ defined as $u =c$ on *A*, satisfies $u \in \text {GSBD}(\Omega )$ and

1.7 $$\begin{aligned} \liminf _{k \rightarrow \infty } \mathcal {F}^p_{\varepsilon _k}(u_k,\Omega ) \ge \mathcal {F}^p(u,\Omega ):= \int _{\Omega } \varphi _{p} (e(u)) \text {d}x + \beta _{p} \mathcal {H}^{n-1}(J_u \cup \partial ^* A), \end{aligned}$$

where $\varphi _p :\mathbb {M}^{n \times n}_{sym} \rightarrow [0,\infty )$ is a 2-homogeneous function and $\beta _p$ is a positive constant. For $p=1$ we have the following explicit form

$$\begin{aligned} \mathcal {F}^1(u,\Omega )= \frac{\pi ^{\frac{n}{2}}}{2}\int _{\Omega } \Big (|e(u)|^2+\frac{1}{2}\text {div}(u)^2\Big )\,\text {d}x +\frac{\pi ^{\frac{n+1}{2}}}{2}\mathcal {H}^{n-1}(J_u), \end{aligned}$$

whenever $$u \in GSBD (\Omega )$$.

For the precise definition of the bulk energy density $\varphi _p$ and of the surface energy density $\beta _p$ we refer to Lemma 4.6.

In proving Theorem 1.1 we addressed two main correlated hurdles. The first difficulty concerns the identification of a limit map *u*. In this respect, we may compare our setting to the existing literature concerning nonlocal approximation of Free Discontinuity problems. In particular, the recent works [28, 35, 39], inspired by the corresponding results on the Mumford-Shah functional [7, 36], provide a nonlocal approximation of a class of Griffith-type functionals in terms of a bulk energy of the form

$$\begin{aligned} \frac{1}{\varepsilon } \int _{\Omega } f \big ( \varepsilon W(e(u)) * \rho _{\varepsilon }\big )\, \textrm{d}x \end{aligned}$$

featuring the convolution of a volume density *W*(*e*(*u*)) for a Sobolev map *u* and a potential *f* whose behavior is similar to that of $\arctan$ in (1.3). The compactness issues in the mentioned articles are overcome due to the presence of an a priori differential structure of the displacement *u*, relying on compactness in $\textrm{GSBD}$ [19, 20]. We remark that such a direct approach is not applicable in our framework, as $u_{\varepsilon }$ are only measurable fields with no control on their symmetric gradient. A similar issue appeared in a discrete fashion in [1, 23] on a deterministic lattice, which indeed proposed a discrete finite-difference approximation of Griffith-type of energies in the spirit of [5, 17, 18, 38] with a finite range of interaction. Their approach to compactness builds upon the construction of $\textrm{GSBD}$-competitors with uniformly bounded energy, which is facilitated by the lack of long-range interactions. This approach allowed again for the application of compactness in $\textrm{GSBD}$. The continuous nature of $\mathcal {F}^{p}_{\varepsilon }$ and its infinite horizon make the adaptation of the strategy of [23] rather challenging.

It turned out that in our setting we can work directly on the sequence $u_{\varepsilon }$, bypassing the modification approach of [23]. Our method relies on Fréchet-Kolmogorov, and thus amounts to prove equi-continuity of translations. This technical step is provided in the proof of Theorem 1.1 and shares common ideas with [3], where the proof of the compactness theorem of [19] was first revisited, avoiding the use of Korn and Korn-Poincaré inequalities. Once again, the lack of a symmetric gradient and the structure of $\mathcal {F}_{\varepsilon }$, which features an extra integration over the directions $\xi \in \mathbb {R}^{n}$, do not allow us to select a preferred basis to control translations, as it was the case in [3]. Nevertheless, the slicing properties of the functionals $\mathcal {F}_{\varepsilon }$ make it possible to control the translations of the maps $\arctan (u_{\varepsilon }(x)\cdot \xi )$ both with respect to *x* and $\xi$. Pointwise convergence out of an exceptional set $A{\subset } \Omega$ is thus recovered by Fréchet-Kolmogorov Theorem.

The next step in the proof of Theorem 1.1 consists in showing that *u* belongs to $\textrm{GSBD}(\Omega )$, yielding the characterization of the domain of the $\Gamma$-limit of $\mathcal {F}^{p}_{\varepsilon }$. Broadly speaking, the non-local nature of the approximating functional $\mathcal {F}^p_\varepsilon$ results in a limiting function space consisting of measurable vector fields that exhibit generalized bounded deformation in a weaker sense. To explain this phenomenon more precisely, recall that $u \in \textrm{GBD}(\Omega )$ if and only if *u* is a measurable vector field and there exists a finite Radon measure $\lambda$ on $\Omega$ such that, for every $\xi \in \mathbb {S}^{n-1}$, the generalized directional variation $\hat{\mu }_u^\xi$ of $u \cdot \xi$, as introduced in [26, Definition 4.10], satisfies:

1.8 $$\begin{aligned} \hat{\mu }^\xi _u(B) \le \lambda (B), \quad \text {for every Borel set } B \subset \Omega . \end{aligned}$$

By a slicing argument, a similar approach to that proposed by M. Gobbino in [31] gives that the limit field $u \in L^{0} (\Omega ; \mathbb {R}^{n})$ satisfies condition (1.8) in an averaged form with respect to $\xi$, namely,

1.9 $$\begin{aligned} \int _{\mathbb {S}^{n-1}} \hat{\mu }^\xi _u(B) \, \textrm{d}\mathcal {H}^{n-1}(\xi ) \le \lambda (B), \quad \text {for every Borel set } B \subset \Omega . \end{aligned}$$

The fact that (1.8) is replaced in our setting by (1.9) is a huge obstacle to establish the structural properties of the limiting function space. In particular, the technique presented in [26] for deriving the GBD-structural properties fails, and we need to rely on the more recent integral geometric approach introduced in [2]. The main difference from the approach in [2] lies in the role of the flatness of $\mathbb {R}^n$, where rescaling and translating the domain preserve straight lines as geodesics, maintaining invariance in the associated GBD-space. Furthermore, the $\textrm{GBD}$-theory developed in [25] provides a quite general method to relate the jump set of *u* to the behavior of codimension one slices, which we are able to adapt to our setting. We remark that such an approach seems not to be successful in a Riemannian manifold. In Propositions 3.15–3.17, we leverage the above features to avoid relying on a Korn-Poincaré-type inequality to estimate the dimensionality of the set on which the jump of all one-dimensional slices of *u* concentrate. Ultimately, we conclude in Theorem 3.1 that the limit map *u* belongs to $\text {GSBD}(\Omega )$, and thus characterize the domain of the $\Gamma$-limit of $\mathcal {F}^{p}_{\varepsilon }$. It is worth noting that, although it is not used in the present paper, this method also allows one to address the case of jump sets with infinite length, which may be of interest for cohesive models obtained as the $\Gamma$-limit of finite-difference approximating functionals, as in [32]. For the detailed definitions of the relevant quantities and the proof of the structural properties, we refer the reader to Sections 2 and 3, respectively.

**An alternative approach.**

After the first version of this paper was submitted as a Preprint on arXiv, a new characterization of GSBD with finite jump was proved in [21], from which it was deduced that our limiting space is GSBD. This characterization was based on controlling the measures $\hat{\mu }^\xi _u$ through finitely many direction $\xi$, which turns out to be equivalent to a control on average. In this new version we propose a totally different perspective, by first deriving fine properties of the limiting space and then proving it coincides with GSBD via some integro-geometrical arguments, hence providing an alternative proof to [21].

Eventually, we obtain the following $\Gamma$-convergence result for the sequence $\mathcal {F}^{p}_{\varepsilon }$ (see Definition 2.1).

### Theorem 1.2

($\Gamma$-convergence) Let $\Omega \subset \mathbb {R}^n$ be open. For every $p\ge 1$ the family of functionals $\{\mathcal {F}^p_\varepsilon \}_{\varepsilon >0}$ $\Gamma$-converges in $L^0(\Omega ;\mathbb {R}^n)$ to the Griffith-type functional $\mathcal {F}^p$ defined as

1.10 $$\begin{aligned} \mathcal {F}^{p} (u, \Omega ):= {\left\{ \begin{array}{ll} \displaystyle \int _{\Omega } \varphi _{p} (e(u)) \text {d}x + \beta _{p} \mathcal {H}^{n-1}(J_u)&  \text {for }u \in \textrm{GSBD}^2(\Omega ),\\ \displaystyle \infty & \text {otherwise in }L^{0} (\Omega ; \mathbb {R}^{n}), \end{array}\right. } \end{aligned}$$

where $\varphi _{p}$ and $\beta _{p}$ are given as in Theorem 1.1.

Combining Theorem 1.1 with the $\Gamma$-convergence of Theorem 1.2, we further deduce convergence of minimisers of $\mathcal {F}_{\varepsilon }^p$ to minimisers of $\mathcal {F}^p$ under suitable Dirichlet boundary conditions (cf. Theorem 5.2).

**Organization of the paper.** The paper is organized as follows. In Section 2, we collect a few preliminary definitions and results. Section 3 is devoted to establish the main properties of the spaces of limiting deformations. Sections 4 and 5 are devoted to the proofs of our compactness, and $\Gamma$-convergence result, respectively.

**Outlook.** The analysis provided in this work offers the opportunity to explore several further research lines. On the one hand, the compactness argument of Theorem 1.1 and the slicing technique of Theorem 1.2 may be further tested against discrete finite-difference approximations on point clouds of vectorial free discontinuity problems, in the spirit of [8, 16, 30] for the Total Variation, the Mumford-Shah, and the perimeter functionals. Such research line is the subject of a preprint which is currently in preparation.

Moreover, the blow-up strategy developed in the proof of Lemma 4.6 appears flexible enough to address nonhomogeneous energies, namely functionals with *x*-dependent bulk densities. In this setting, no additional obstruction arises at the level of compactness, as soon as the inhomogeneous bulk part is controlled from below by the corresponding homogeneous density. Under this assumption, the compactness argument carries over without substantial modifications, and the localization underlying the blow-up procedure remains applicable.

On the other hand, the use of a slicing argument for the study of the variational limit restricts the choice of the approximating sequence $\mathcal {F}_{\varepsilon }$, which in turn determines the class of admissible densities $\varphi _{p}$ in (1.10). The study of more general approximations and of integral representation formulas in the spirit of [1, 22, 23] will be the subject of future investigation. A possible strategy to generalize our result to the case of arbitrary Lamé constants could be to rely on the presence of further discrete-divergence terms, along the lines of [1, 23]. The extension of our compactness result and of our identification of the limiting domain for such a model would be possible without any change to the structure of the current proof. The associated Gamma-convergence analysis, on the other hand, deserves the development of a novel representation theory.

## Definitions and notation

Recall the functionals $\mathcal {F}^p_\varepsilon$ and $F_{\varepsilon ,\xi }$ defined in the introduction. Given $A \subset \mathbb {R}$, we further set

$$\begin{aligned} F_{\varepsilon }(u,A):=\frac{1}{\varepsilon } \int _{A} \arctan \left( \frac{( u(t+\varepsilon )-u(t))^2}{\varepsilon } \right) \text {d}t, \end{aligned}$$

for every measurable function $u:B \subset \mathbb {R} \rightarrow \mathbb {R}$ such that $A \subset B \cap (B-\varepsilon )$. For the sequel it is convenient to introduce the following class of Mumford-Shah and Griffith functionals. For $\gamma >0$, the former reads as

$$\begin{aligned} \mathcal{M}\mathcal{S}_{\gamma }(u,\Omega ) :={\left\{ \begin{array}{ll} \gamma \int _{\Omega } |\nabla u(x)|^2 \text {d}x+ \mathcal {H}^{n-1}(J_u), \quad u \in \text {GSBV}(\Omega ),\\ +\infty , \qquad u \in L^1_{loc}(\Omega )\setminus \text {GSBV}(\Omega ). \end{array}\right. } \end{aligned}$$

For $\lambda >0$, we define the Griffith functional by

$$\begin{aligned} \mathcal {G}_{\lambda }(u,\Omega ) :={\left\{ \begin{array}{ll} \lambda \int _{\Omega } | e(u)(x)|^2 \text {d}x+ \mathcal {H}^{n-1}(J_u), \quad u \in \text {GSBD}(\Omega ),\\ \infty , \qquad u \in L^0(\Omega ;\mathbb {R}^n)\setminus \text {GSBD}(\Omega ). \end{array}\right. } \end{aligned}$$

For every $\xi \in \mathbb {R}^{n} \setminus \{0\}$, every $x \in \mathbb {R}^n$, and every $E\subset \mathbb {R}^{n}$, we define

$$\begin{aligned} E^{\xi }_{x}:= \{ t \in \mathbb {R}: \, x + t\xi \in E\}\,. \end{aligned}$$

For $u :E \rightarrow \mathbb {R}^{n}$ we define $\hat{u}^{\xi }_{x} :E^{\xi }_{x} \rightarrow \mathbb {R}$ as

$$ \hat{u}^{\xi }_{x} (t) := u(x + t\xi ) \cdot \xi . $$

For $v :E \rightarrow \mathbb {R}$, we denote by $v^{\xi }_{x}:E^{\xi }_{x} \rightarrow \mathbb {R}$ the function $v^{\xi }_{x} (t):= v(x + t\xi )$. For $A \subset \mathbb {R}$ open and $v :A \rightarrow \mathbb {R}$ measurable, we set $J^{1}_{v}:=\{t \in J_{u} \cap A: \,| v^{+}(t) - v^{-}(t)| > 1 \}$. We also define $\Pi ^\xi :=\{y \in \mathbb {R}^n: y \cdot \xi = 0 \}$ the plane orthogonal to $\xi$, and $\pi _\xi : \mathbb {R}^n \rightarrow \Pi ^\xi$ the projection on $\Pi ^\xi$.

With this notation at hands, we recall the definition of $\text {GBD}$ and $\text {GSBD}$, namely, the spaces of functions with generalized bounded deformation and generalized special bounded deformation, respectively (see [26]).

### Definition 2.1

(The spaces $\text {GBD}/\text {GSBD}/\text {GSBD}^2$) We say that an $\mathcal {L}^n$-measurable function $u :\Omega \rightarrow \mathbb {R}^n$ belongs to the space $\text {GBD}(\Omega )$ if there exists a finite Borel measure $\lambda$ on $\Omega$ such that for every $\xi \in \mathbb {S}^{n-1}$ and for $\mathcal {H}^{n-1}$-a.e. $y \in \Pi ^\xi$ the function $\hat{u}_y^\xi \in \text {BV}_{loc}(\Omega _y^\xi )$ and

2.1 $$\begin{aligned} \int _{\Pi ^\xi } |D \hat{u}_y^\xi |( B_y^\xi \setminus J^1_{\hat{u}_y^\xi })+\mathcal {H}^0( B_y^\xi \cap J^1_{\hat{u}_y^\xi }) \text {d}\mathcal {H}^{n-1}(y) < \lambda (B), \end{aligned}$$

whenever $B \subset \Omega$ is a Borel set. Moreover, we say that $u \in \text {GSBD}(\Omega )$ if $u \in \text {GBD}(\Omega )$ and $\hat{u}_y^\xi \in \text {SBV}_{loc}(\Omega _y^\xi )$ for every $\xi \in \mathbb {S}^{n-1}$ and for $\mathcal {H}^{n-1}$-a.e. $y \in \Pi ^\xi$.

Eventually, we also denote by $GSBD^2(\Omega )$ the space defined as

$$ \text {GSBD}^2(\Omega ):= \{u \in \text {GSBD}(\Omega ): \, e(u) \in L^2(\Omega ;\mathbb {M}^n_{sym}), \ \mathcal {H}^{n-1}(J_u) < \infty \}. $$

The proof of the following measurability property is postponed to Section 3.

### Lemma 2.2

Let $u :\Omega \rightarrow \mathbb {R}^n$ be $\mathcal {L}^{n}$-measurable. Assume that for $\mathcal {H}^{n-1}$-a.e. $\xi \in \mathbb {S}^{n-1}$, for $\mathcal {H}^{n-1}$-a.e. $y \in \Pi ^{\xi }$, the function $\hat{u}^{\xi }_{y}$ belongs to $\textrm{BV}_{loc} (\Omega ^{\xi }_{y})$. Then, for every open set $U \subset \Omega$ the map

2.2 $$\begin{aligned} (x,\xi ) \mapsto | D\hat{u}^{\xi }_{x}| (U^{\xi }_{x} \setminus J^{1}_{\hat{u}^{\xi }_{x}}) + \mathcal {H}^{0} (U^{\xi }_{x} \cap J^{1}_{\hat{u}^{\xi }_{x}}) \end{aligned}$$

is $(\mathcal {L}^n \otimes \mathcal {H}^{n-1})$-measurable on $\Omega \times \mathbb {S}^{n-1}$. In particular, for every open set $U \subset \Omega$ we have that the map

$$\begin{aligned} \xi \mapsto \int _{\Pi ^\xi } |D \hat{u}_y^\xi |( U_y^\xi \setminus J^1_{\hat{u}_y^\xi })+\mathcal {H}^0( U_y^\xi \cap J^1_{\hat{u}_y^\xi }) \text {d}\mathcal {H}^{n-1}(y) \end{aligned}$$

is $\mathcal {H}^{n-1}$-measurable on $\mathbb {S}^{n-1}$.

Next, we recall a measurability property originally stated in [26, Lemma 3.6].

### Lemma 2.3

Let $v :\Omega \rightarrow \mathbb {R}$ be $\mathcal {L}^{n}$-measurable and let $\xi \in \mathbb {S}^{n-1}$. Assume that for $\mathcal {H}^{n-1}$-a.e. $y \in \Pi ^{\xi }$, the function $v^{\xi }_{y}$ belongs to $\textrm{BV}_{loc} (\Omega ^{\xi }_{y})$. Then, for every Borel set $B \subset \Omega$ the map

$$\begin{aligned} y \mapsto | Dv^{\xi }_{y}| (B^{\xi }_{y} \setminus J^{1}_{v^{\xi }_{y}}) + \mathcal {H}^{0} (B^{\xi }_{y} \cap J^{1}_{v^{\xi }_{y}}) \end{aligned}$$

is $\mathcal {H}^{n-1}$-measurable on $\Pi ^\xi$.

We are now in position to define the space $\textrm{GBV}^\mathcal {E}{}(\Omega ;\mathbb {R}^n)$.

### Definition 2.4

(The space $\text {GBV}^\mathcal {E}$) We say that a $\mathcal {L}^n$-measurable function $u :\Omega \rightarrow \mathbb {R}^n$ belongs to the space $\text {GBV}^{\mathcal {E}}(\Omega ;\mathbb {R}^n)$ if for $\mathcal {H}^{n-1}$-a.e. $\xi \in \mathbb {S}^{n-1}$ and for $\mathcal {H}^{n-1}$-a.e. $y \in \Pi ^\xi$ the function $\hat{u}_y^\xi \in \text {BV}_{loc}(\Omega _y^\xi )$ and

2.3 $$\begin{aligned} \int _{\mathbb {S}^{n-1}} \bigg ( \int _{\Pi ^\xi } |D \hat{u}_y^\xi |( \Omega _y^\xi \setminus J^1_{\hat{u}_y^\xi })+\mathcal {H}^0( \Omega _y^\xi \cap J^1_{\hat{u}_y^\xi }) \text {d}\mathcal {H}^{n-1}(y) \bigg ) \text {d}\mathcal {H}^{n-1}(\xi ) <\infty . \end{aligned}$$

Moreover, we say that $u \in \text {GSBV}^{\mathcal {E}}(\Omega ;\mathbb {R}^n)$ if $u \in \text {GBV}^{\mathcal {E}}(\Omega ;\mathbb {R}^n)$ and $\hat{u}_y^\xi \in \text {SBV}_{loc}(\Omega _y^\xi )$ for $\mathcal {H}^{n-1}$-a.e. $\xi \in \mathbb {S}^{n-1}$ and for $\mathcal {H}^{n-1}$-a.e. $y \in \Pi ^\xi$.

### Remark 2.5

Observe that the space $\text {GBV}^{\mathcal {E}}(\Omega ;\mathbb {R}^n)$ introduced in Definition 2.4 does not coincide a priori with $\text {GBD}(\Omega )$ introduced in [26, Definition 4.1]. This is because the control in (2.3) is not pointwise in $\xi$ but only in average with respect to the -measure. Nevertheless, we will show in Section 3.2 that actually $\text {GSBV}^{\mathcal {E}}(\Omega ;\mathbb {R}^n)=\text {GSBD}(\Omega )$.

In view of the measurability property contained in Lemma 2.3 we provide the following definition.

### Definition 2.6

Let $\Omega \subset \mathbb {R}^{n}$ open and let $u :\Omega \rightarrow \mathbb {R}^{n}$ be $\mathcal {L}^{n}$-measurable. Then for every $\xi \in \mathbb {S}^{n-1}$ we define the Borel regular measure $\hat{\mu }^\xi _u$ in $\Omega$ as follows. We define $\hat{\mu }^\xi _u$ on $\Omega$ as the unique Borel regular measure on $\Omega$ that satisfies for every open set $U \subset \Omega$

$$\begin{aligned} \hat{\mu }^{\xi }_{u} (U) := \int _{\Pi ^{\xi }}| D\hat{u}^{\xi }_{y}| (U^{\xi }_{y} \setminus J^{1}_{\hat{u}^{\xi }_{y} } ) + \mathcal {H}^{0} (U^{\xi }_{y} \cap J^{1}_{\hat{u}^{\xi }_{y} }) \, \textrm{d}\mathcal {H}^{n-1} (y), \end{aligned}$$

whenever for $\mathcal {H}^{n-1}$-a.e. $y \in \Pi ^{\xi }$ the function $\hat{u}^{\xi }_{y}$ belongs to $\textrm{BV}_{loc}(U^{\xi }_{y})$, while $\hat{\mu }^\xi _u(U)=\infty$ otherwise.

### Remark 2.7

Since the hypothesis of De Giorgi-Letta’s Theorem [4] are fulfilled, the extension of the set function $U \mapsto \hat{\mu }^\xi _u(U)$ to a Borel regular measure on $\Omega$ is uniquely guaranteed by the formula

$$\begin{aligned} \hat{\mu }^\xi _u(B) := \inf \{ \hat{\mu }^\xi _{u}(U) : B \subset U, \ U \subset \Omega \text { open} \}. \end{aligned}$$

### Remark 2.8

In view of (2.3), if $u \in \textrm{GBV}^{\mathcal {E}} (\Omega )$ we have that $\hat{\mu }^{\xi }_u \in \mathcal {M}^{+}_{b} (\Omega )$ for $\mathcal {H}^{n-1}$-a.e. $\xi \in \mathbb {S}^{n-1}$. Moreover, for such $\xi$, we can make use of the measurability property contained in Lemma 2.3 to infer

$$ \hat{\mu }^\xi _u(B) = \int _{\Pi ^{\xi }}| D\hat{u}^{\xi }_{y}| (B^{\xi }_{y} \setminus J^{1}_{\hat{u}^{\xi }_{y} } ) + \mathcal {H}^{0} (B^{\xi }_{y} \cap J^{1}_{\hat{u}^{\xi }_{y} }) \, \textrm{d}\mathcal {H}^{n-1} (y), $$

for every Borel set $B \subset \Omega$.

### Definition 2.9

For $p \ge 1$, $u \in \textrm{GBV}^{\mathcal {E}} (\Omega )$, and $\xi \in \mathbb {S}^{n-1}$ such that $\hat{\mu }^{\xi }_{u} \in \mathcal {M}^{+}_{b} (\Omega )$, we define $\hat{\mu }_{u,p}$ on $\Omega$ as the unique Borel regular measure on $\Omega$ that satisfies

2.4 $$\begin{aligned} \hat{\mu }_{u,p} (U):= \sup _{\mathscr {B}} \sum _{B \in \mathscr {B}} \bigg (\int _{\mathbb {S}^{n-1}} \hat{\mu }^{\xi }_{u}(B)^{p}\, \textrm{d}\mathcal {H}^{n-1}(\xi ) \bigg )^{\frac{1}{p}} \qquad \text {for }U\subset \Omega \text { open,} \end{aligned}$$

where $\mathscr {B}$ denotes any finite family of pairwise disjoints open balls contained in *U*.

Observe that for $p=1$ the following explicit representation of $\hat{\mu }_{u,1}$ holds true

2.5 $$\begin{aligned} \hat{\mu }_{u,1} (U) = \int _{\mathbb {S}^{n-1}} \hat{\mu }^{\xi }_{u} (U)\, \textrm{d}\mathcal {H}^{n-1}(\xi )\qquad \text {for }U\subset \Omega \text { open}. \end{aligned}$$

### Remark 2.10

We observe that, by virtue of Jensen’s inequality, it holds true that

$$ {\hat{\mu }_{u,1}} \le \mathcal {H}^{n-1}(\mathbb {S}^{n-1})^{\frac{p-1}{p}} {\hat{\mu }_{u,p}}, $$

in the sense of measures for every $p \ge 1$.

### Remark 2.11

Also in this case, by De Giorgi-Letta’s Theorem [4] the extension of the set function $U \mapsto \hat{\mu }_{u,p}(U)$ to a Borel regular measure on $\Omega$ is uniquely guaranteed by

$$ \begin{aligned} \hat{\mu }_{u,p}(B) := \inf \{ \hat{\mu }_{u,p}(U) : B \subset U, \ U \subset \Omega \text { open} \}. \end{aligned} $$

The above formula immediately gives also the uniqueness of the extension to a Borel regular measure on $\Omega$.

We conclude this section with a remark concerning the definition of the functionals $\mathcal {F}^p_\varepsilon$ as well as the definition of the measures $\hat{\mu }_{u,p}$.

### Remark 2.12

We observe that in (1.4) and (2.4) we can equivalently take the supremum over any family of countable pairwise disjoint open balls and the result would be the same. In addition, all the results as well as their proofs remain unchanged if we replace $\mathscr {B}$ with any class made of finitely many pairwise disjoint convex and open sets.

We also remark that the functional (1.4) could be replaced by

$$\begin{aligned} \mathcal {F}'^p_\varepsilon (u,\Omega ):=\sup _{ \mathscr {B}} \sum _{B \in \mathscr {B}}\bigg (\int _{\frac{B-B}{\varepsilon }}F_{\varepsilon ,\xi }(u,B)^p e^{-|\xi |^{2}}\, d\xi \bigg )^\frac{1}{p } \end{aligned}$$

without any substantial change in the proofs that follow. In particular, with this choice we would have exactly $\mathcal {F}'^1_\varepsilon (u,B)=\mathcal {F}_\varepsilon (u,B)$ for every open ball $B \subset \mathbb {R}^n$.

## Structure of the space $\text {GBV}^{\mathcal {E}}$

The present section addresses the study of the space $\textrm{GBV}^{\mathcal {E}}(\Omega ;\mathbb {R}^n)$ introduced in Definition 2.4. In particular, we focus on the structure of functions $u \in \textrm{GSBV}^{\mathcal {E}}(\Omega ,\mathbb {R}^n)$. As a byproduct, we prove the following identification Theorem.

### Theorem 3.1

Let $\Omega \subset \mathbb {R}^{n}$ open. A measurable function $u :\Omega \rightarrow \mathbb {R}^n$ belongs to $\textrm{GSBD}(\Omega )$ if and only if $u \in \textrm{GSBV}^{\mathcal {E}}(\Omega ;\mathbb {R}^n)$.

### Remark 3.2

It is worth noticing that in Theorem 3.1 we do not assume $\mathcal {H}^{n-1}(J_u) < \infty$.

In the next subsection, we discuss some measurability issues which justify formula (2.3) in Definition 2.4.

### Preliminaries

We start with a technical proposition for one-dimensional functions. We introduce the class $\mathcal {T}$ of all functions $\tau \in C^{1}(\mathbb {R})$ such that $-\frac{1}{2} \le \tau \le \frac{1}{2}$ and $0 \le \tau '\le 1$. We recall that, given an open set $I \subset \mathbb {R}$ and given $(\tau _j) \subset C^1(I)$, $\tau \in C^1(I)$, we have $\tau _j \rightarrow \tau$ in $C^1_{loc}(I)$ if and only if for every open set $U \Subset I$ it holds $\Vert \tau _j-\tau \Vert _{L^\infty (U)} + \Vert \tau '_j - \tau '\Vert _{L^\infty (U)} \rightarrow 0$ as $j \rightarrow \infty$.

#### Proposition 3.3

Let $I \subset \mathbb {R}$ be open and let $u \in \textrm{BV}_{loc}(I)$. Let furthermore $\hat{\mathcal {T}} \subset \mathcal {T}$ be any countable set which is dense with respect to the $C^1_{loc}$-topology. For every *U* open subset of *I*, it holds

3.1 $$\begin{aligned} |Du|(U \setminus J^1_u) + \mathcal {H}^0(U \cap J^1_u) = \sup _{k \in \mathbb {N}} \sup \sum _{i=1}^{k} | D(\tau _{i} (u)) |(U_{i})\,, \end{aligned}$$

where the second supremum is taken over all the families $\tau _{1}, \ldots \tau _{k} \in \hat{\mathcal {T}}$ and all the families of pairwise disjoint open subsets $U_{1}, \ldots , U_{k}$ of *U*.

#### Proof

Since $u \in \textrm{BV}_{loc}(I)$, for every $U \Subset I$ open and compactly contained in *I*, the condition (a) of [26, Theorem 3.5] is satisfied. Therefore, we can apply [26, Theorem 3.8] to the one-dimensional function  to deduce that (3.1) holds true for every open set $U' \subset U$ if we replace $\hat{\mathcal {T}}$ with the entire family $\mathcal {T}$. In order to pass from a compactly contained open set *U* to the whole of *I*, it is enough to consider a sequence $U_k \Subset I$ with $U_k \nearrow I$ and notice that

$$ |Du|(U_k \setminus J^1_u) + \mathcal {H}^0(U_k \cap J^1_u) \nearrow |Du|(I \setminus J^1_u) + \mathcal {H}^0(I \cap J^1_u), \ \ \text { as }k \rightarrow \infty . $$

In order to conclude the proof of formula (3.1) we need to show that it is enough to consider the supremum on the smaller family $\hat{\mathcal {T}}$. To this purpose we simply notice that for every $U \Subset I$, being $u \in \textrm{BV}(U)$, we have $\Vert u\Vert _{L^\infty (U)} \le m$. Hence, by applying the chain rule for $\textrm{BV}$-function (see for instance [4]), if $(\tau _j) \subset \hat{\mathcal {T}}$ is such that $\tau _j \rightarrow \tau$ in $C^1_{loc}(\mathbb {R})$, then

$$ |D(\tau _j(u) - \tau (u))|(U) \le \Vert \tau _j -\tau \Vert _{C^1((-m,m))} \, |Du|(U) \rightarrow 0, \ \ \text { as }j \rightarrow \infty . $$

With this information at hand we infer that the double supremum in the right hand-side of (3.1) does not change when restricted to $\hat{\mathcal {T}}$. $\square$

We are now in position to prove Lemma 2.2.

#### Proof of Lemma 2.2

The assumptions of the lemma are equivalent to: for $\mathcal {H}^{n-1}$-a.e. $\xi \in \mathbb {S}^{n-1}$, for $\mathcal {L}^{n}$-a.e. $x \in \Omega$, the function $\hat{u}^{\xi }_{x}$ belongs to $\textrm{BV}_{loc} (\Omega ^{\xi }_{x})$.

We claim that for every open set $U \subset \Omega$ and every $\tau \in \mathcal {T}$ the map $(x,\xi ) \mapsto | D\tau (\hat{u}^\xi _x) |(U^\xi _x)$ is $(\mathcal {L}^n \otimes \mathcal {H}^{n-1})$-measurable on $\Omega \times \mathbb {S}^{n-1}$. To show the claim, consider a countable dense subset $D \subset C^1_c(\mathbb {R})$ in the $C^1_{loc}$-topology. Notice that, for every $f \in L^1_{loc}(\mathbb {R})$ and every $I \subset \mathbb {R}$ open, we have

3.2 $$\begin{aligned} | D f |(I) = \sup _{\begin{array}{c} \varphi \in C^1_c(I) \\ \Vert \varphi \Vert _{L^\infty } \le 1 \end{array}} \int _{\mathbb {R}} f \, D\varphi \, \text {d}t = \sup _{\begin{array}{c} \varphi \in C^1_c(I) \cap D \\ \Vert \varphi \Vert _{L^\infty } \le 1 \end{array}} \int _{\mathbb {R}} f \, D\varphi \, \text {d}t. \end{aligned}$$

Now consider a sequence of open sets $U'_k \Subset U$ with $U'_k \nearrow U$, and consider a sequence of functions $v_k \in C^1_c(U)$ with $0 \le v_k \le 1$ while $v_k=1$ on $U'_k$. Notice that, for every $\varphi \in C^1_c(\mathbb {R})$, the map $(x,\xi ) \mapsto \int _{\mathbb {R}} \tau (\hat{u}^\xi _x) \, D((v_k)^\xi _x \varphi ) \, \text {d}t$ is $(\mathcal {L}^n \otimes \mathcal {H}^{n-1})$-measurable (for instance by virtue of Fubini’s Theorem). By formula (3.2), we have for every $(x, \xi ) \in \Omega \times \mathbb {S}^{n-1}$ that

$$ |D\tau (\hat{u}^\xi _x)|((U'_k)^\xi _x) \le h_k(x,\xi ):= \! \! \! \! \! \!\sup _{\begin{array}{c} \varphi \in C^1_c(\mathbb {R}) \cap D \\ \Vert \varphi \Vert _{L^\infty } \le 1 \end{array}} \int _{\mathbb {R}} \tau (\hat{u}^\xi _x) \, D((v_k)^\xi _x \varphi ) \, \text {d}t \le |D\tau (\hat{u}^\xi _x)|(U^\xi _x). $$

Notice that, since the countable supremum of measurable functions is a mesurable function, $h_k(x,\xi )$ is $(\mathcal {L}^n \otimes \mathcal {H}^{n-1})$-measurable. By the monotone property of measures, we know that $|D\tau (\hat{u}^\xi _x)|((U'_k)^\xi _x) \nearrow |D\tau (\hat{u}^\xi _x)|(U^\xi _x)$ for every $(x,\xi ) \in \Omega \times \mathbb {S}^{n-1}$ as $k \rightarrow \infty$, meaning that $h_k(x,\xi ) \rightarrow |D\tau (\hat{u}^\xi _x)|(U^\xi _x)$ for every $(x,\xi ) \in \Omega \times \mathbb {S}^{n-1}$ as $k \rightarrow \infty$. Thanks to the fact that pointwise limits of sequences of measurable functions are measurable functions, the claim follows.

By combining the previous claim with Proposition 3.3, we deduce that the map in (2.2) is the countable supremum of measurable maps, therefore, it is measurable. The proof is thus concluded. $\square$

We discuss here the measurability of $\xi \mapsto \hat{\mu }^{\xi }_{u} (B)$ for *B* Borel subset of $\Omega$.

#### Proposition 3.4

Let $\Omega \subset \mathbb {R}^{n}$ open and let $u :\Omega \rightarrow \mathbb {R}^{n}$ be $\mathcal {L}^{n}$-measurable and such that for $\mathcal {H}^{n-1}$-a.e. $\xi \in \mathbb {S}^{n-1}$ and $\mathcal {H}^{n-1}$-a.e. $y \in \Pi ^{\xi }$ the function $\hat{u}^{\xi }_{y}$ belongs to $\textrm{BV}_{loc}(\Omega ^{\xi }_{y})$. For every $\xi \in \mathbb {S}^{n-1}$ and for every *U* open subset of $\Omega$, it holds

3.3 $$\begin{aligned} \hat{\mu }^{\xi }_{u} (U) = \sup _{k \in \mathbb {N}} \sup \sum _{i=1}^{k} | D_{\xi } (\tau _{i} (u\cdot \xi )) |(U_{i})\,, \end{aligned}$$

where the second supremum is taken over all the families $\tau _{1}, \ldots \tau _{k} \in \mathcal {T}$ and all the families of pairwise disjoint open subsets $U_{1}, \ldots , U_{k}$ of *U*.

#### Proof

Whenever $\xi \in \mathbb {S}^{n-1}$ and $U \subset \Omega$ are such that $\hat{\mu }^{\xi }_{u}(U) <\infty$, equality (3.3) holds in view of [26, Theorem 3.8]. It remains to consider the case $\hat{\mu }^{\xi }_{u} (U) = \infty$. For simplicity of notation, we denote by $\eta$ the set function given by the right-hand side of (3.3). By contradiction, assume that $\eta (U)<\infty$. In particular, for every $\tau \in \mathcal {T}$ we have

3.4 $$\begin{aligned} |D_{\xi } \tau (u\cdot \xi )|(U)\le \eta (U) < \infty \,. \end{aligned}$$

Hence, $D_{\xi } \tau (u\cdot \xi ) \in \mathcal {M}_{b} (U)$. By a Carathéodory construction using $\eta$ as Gauge function and the family of all open balls contained in *U* as set of generators, we obtain a Borel regular measure $\lambda$ on *U*, with $\lambda _{\delta }$ as approximating measure for $\delta >0$ (see, e.g., [29, Section 2.10.1]). For every $V \Subset U$, for every $\delta >0$ small enough, and for every covering $\mathcal {G}$ of *V* made of open balls in $\mathbb {R}^{n}$ with diameter smaller than $\delta$, we may assume that $\bigcup _{A \in \mathcal {G}} A \subset U$. By Besicovitch covering theorem, there exists a dimensional constant $c(n)>0$ and $\mathcal {G}_{1}, \ldots , \mathcal {G}_{c(n)}$ disjoint countable subfamilies of $\mathcal {G}$ such that

$$\begin{aligned} V \subset \bigcup _{i=1}^{c(n)} \bigcup _{A \in \mathcal {G}_{i}} A\,. \end{aligned}$$

In particular, we have that $\lambda _{\delta } (V) \le c(n) \eta (U)$, which yields the inequality $\lambda (V) \le c(n) \eta (U)$ for every $V \Subset U$. Taking the limit $V \nearrow U$ we conclude that $\lambda$ is a positive bounded Radon measure on *U*. By using (3.4) it is not difficult to show that actually for every Borel set $B \subset U$ we have that $|D_{\xi } \tau (u\cdot \xi )| (B) \le \lambda (B)$ whenever $\tau \in \mathcal {T}$. Thus, we are in a position to apply [26, Theorem 3.5] to deduce that $\hat{\mu }^{\xi }_{u} (U) \le \lambda (U) <\infty$, which is a contradiction. Thus, it must be $\eta (U) = \infty$ and equality (3.3) is satisfied. $\square$

#### Corollary 3.5

Under the assumptions of Proposition 3.4, for every open set $U \subset \Omega$ the map $\xi \mapsto \hat{\mu }^{\xi }_{u} (U)$ is lower-semicontinuous on $\mathbb {S}^{n-1}$.

#### Proof

We notice that equality (3.3) holds for every $U \subset \Omega$ open, for every $\xi \in \mathbb {S}^{n-1}$. In particular, the right-hand side of (3.3) is lower-semicontinuous w.r.t. $\xi \in \mathbb {S}^{n-1}$ for fixed $U\subset \Omega$ open, hence $\xi \mapsto \hat{\mu }^{\xi }_{u} (U)$ is also lower-semicontinuous. $\square$

Thanks to Corollary 3.5, the integral (2.3) in Definition 2.4 is now justified. We further notice that $\hat{\mu }_{u,1}$ can be extended to a finite Radon measure on $\Omega$, that we still denote by $\hat{\mu }_{u,1}$.

#### Remark 3.6

We point out that whenever $u \in \textrm{GBV}^{\mathcal {E}} (\Omega ;\mathbb {R}^n)$, the integral formula (2.5) can be extended also for $K \subset \Omega$ compact. Indeed, it is enough to take a sequence $U_{k}$ of open subsets of $\Omega$ such that $K = \bigcap _{k \in \mathbb {N}} U_{k}$. Then, for every $k \in \mathbb {N}$ we have that

$$\begin{aligned} \hat{\mu }_{u,1} \bigg (\bigcap _{j=1}^{k} U_{j} \bigg ) = \int _{\mathbb {S}^{n-1}} \hat{\mu }^\xi _{u} \bigg (\bigcap _{j=1}^{k} U_{j} \bigg ) \, \text {d}\mathcal {H}^{n-1}(\xi )\,. \end{aligned}$$

Passing to the limit as $k\rightarrow \infty$, by dominated convergence (recall that $\hat{\mu }^{\xi }_{u} (\Omega ) \in L^{1}(\mathbb {S}^{n-1})$) we deduce that

$$\begin{aligned} \hat{\mu }_{u,1} (K ) := \int _{\mathbb {S}^{n-1}} \hat{\mu }^\xi _{u} ( K ) \, \text {d}\mathcal {H}^{n-1}(\xi )\,. \end{aligned}$$

### Proof of Theorem 3.1

In this section we prove that if $u \in \text {GSBV}^{\mathcal {E}}(\Omega ;\mathbb {R}^n)$ then $u \in \text {GSBD}(\Omega )$. Such implication is a consequence of the following two theorems, concerning the structure properties of functions in $\textrm{GSBV}^{\mathcal {E}} (\Omega ;\mathbb {R}^n)$.

#### Theorem 3.7

Let $u \in \textrm{GBV}^{\mathcal {E}}(\Omega ;\mathbb {R}^n)$. Then for $\mathcal {H}^{n-1}$-a.e. $\xi \in \mathbb {S}^{n-1}$ we have

3.5 $$\begin{aligned} (J_u)^\xi _y = J_{\hat{u}^\xi _y}, \quad \text {for }\mathcal {H}^{n-1}\text {-a.e. } y \in \Pi ^\xi \end{aligned}$$

#### Theorem 3.8

Let $u \in \text {GBV}^{\mathcal {E}}(\Omega ;\mathbb {R}^n)$. Then there exists $e(u) \in L^1(\Omega ;\mathbb {M}_{\text {sym}}^{n \times n})$ such that

$$\begin{aligned} \mathop {\mathrm {ap-\lim }}\limits _{y \rightarrow x} \frac{(u(y)-u(x)-e(u)(x)(y-x))\cdot (y-x)}{|y-x|^2}=0 \end{aligned}$$

holds for a.e. $x \in \Omega$. Moreover, for a.e. $\xi \in \mathbb {R}^n \setminus \{0\}$ and for $\mathcal {H}^{n-1}$-a.e. $y \in \Pi ^\xi$ we have

3.6 $$\begin{aligned} e(u)_y^\xi \xi \cdot \xi = \nabla \hat{u}^\xi _y \quad \mathcal {L}^1\text {-a.e. on } \Omega _y^\xi . \end{aligned}$$

Postponing the proofs of Theorems 3.7 and 3.8 to Subsections 3.2.1 and 3.2.2, respectively, we conclude this section showing how to exploit them to prove Theorem 3.1.

#### Proof of Theorem 3.1

The only non trivial implication is $u \in \textrm{GSBV}^{\mathcal {E}}(\Omega ;\mathbb {R}^n)$ implies $u \in \textrm{GSBD}(\Omega )$. From the very definition of $\text {GSBD}(\Omega )$ we need to show the existence of a finite Borel measure $\lambda$ on $\Omega$ such that for every $\xi \in \mathbb {S}^{n-1}$

3.7 $$\begin{aligned}&\hat{u}^\xi _y \in \text {SBV}_{loc}(\Omega ^\xi _y), \quad \text {for }\mathcal {H}^{n-1}\text {-a.e. }y \in \Pi ^\xi \end{aligned}$$

3.8 $$\begin{aligned}&\hat{\mu }^\xi _u(B) \le \lambda (B), \quad \text {for every }B \subset \Omega \text { Borel}. \end{aligned}$$

By combining Theorem 3.7 together with the countably $(n-1)$-rectifiability of $J_u$ (see [27]), we make use of the Area Formula to infer for $\mathcal {H}^{n-1}$-a.e. $\xi \in \mathbb {S}^{n-1}$ that for every Borel set $B \subset \Omega$

$$\begin{aligned} \int _{\Pi ^\xi }|D^j\hat{u}^\xi _y|(B^\xi _y \setminus J^1_{\hat{u}^\xi _y}) + \mathcal {H}^0(B^\xi _y \cap J^1_{\hat{u}^\xi _y}) \, \text {d}\mathcal {H}^{n-1}(y) = \int _{B \cap J_u} (|[u \cdot \xi ]| \wedge 1)\,|\nu _u \cdot \xi | \, \text {d}\mathcal {H}^{n-1}, \end{aligned}$$

where $D^j$ denotes the jump part of the distributional derivative. In particular, there exists a dimensional constant $0< c_n <1$ such that for every $v \in \mathbb {R}^n$ and $\nu \in \mathbb {S}^{n-1}$

$$ c_n(|v| \wedge 1) \le \int _{\mathbb {S}^{n-1}}(|v \cdot \xi | \wedge 1)\,|\nu \cdot \xi | \, \text {d}\mathcal {H}^{n-1}(\xi ) \le |v| \wedge 1. $$

In addition, by Theorem 3.8 we have also for $\mathcal {H}^{n-1}$-a.e. $\xi \in \mathbb {S}^{n-1}$ and for every Borel set $B \subset \Omega$

$$\begin{aligned} \int _{\Pi ^\xi } |\nabla \hat{u}^\xi _y|(B^\xi _y) \, \text {d}\mathcal {H}^{n-1}(y)&= \int _{\Pi ^\xi } \bigg ( \int _{B^\xi _y} |e(u)^\xi _y\xi \cdot \xi |\,\text {d}t \bigg )\text {d}\mathcal {H}^{n-1}(y) \\&= \int _B |e(u)\xi \cdot \xi | \, \textrm{d}x. \end{aligned}$$

Therefore, since $\hat{\mu }_{u,1}$ is a finite measure (recall that, by Theorem 3.8, also $|e(u)| \in L^1(\Omega )$), the Borel measure  is finite and for $\mathcal {H}^{n-1}$-a.e. $\xi \in \mathbb {S}^{n-1}$ we have

3.9 $$\begin{aligned} \hat{\mu }^\xi _u(B) \le \lambda (B), \quad \text {for every }B \subset \Omega \text { Borel}. \end{aligned}$$

In order to get the full conditions (3.7) and (3.8) for every $\xi \in \mathbb {S}^{n-1}$, given $\xi \in \mathbb {S}^{n-1}$, we consider $\xi _k \rightarrow \xi$ such that each $\xi _k$ satisfies (3.7), (3.8) and by virtue of Corollary 3.5 we infer that

$$ \hat{\mu }^\xi _u(U) \le \liminf _{k} \hat{\mu }^{\xi _k}_u(U) \le \lambda (U), \quad \text {for every }U \subset \Omega \text { open}. $$

Since $\lambda$ is a finite Borel measure, the above inequality can be further extended to every Borel set $B \subset \Omega$ by outer regularity. This proves (3.8). In order to verify the validity of (3.7) we notice that, from (3.9) and by the definition of $\text {GSBV}^{\mathcal {E}}(\Omega ;\mathbb {R}^n)$, we know that for a dense set of $\xi \in \mathbb {S}^{n-1}$ conditions (3.7) and (3.8) hold true. From [26, Theorem 3.5], for every such $\xi$ we have

3.10 $$\begin{aligned} |D \tau (u \cdot \xi )|(B) \le \lambda (B), \quad \text {for every }B \subset \Omega \text { Borel}, \end{aligned}$$

whenever $\tau \in \mathcal {T}$. By exploiting (3.10) and the lower semi-continuity of $\xi \mapsto |D \tau (u \cdot \xi )|(U)$ for every open set $U \subset \Omega$, we argue as before and show that actually (3.10) holds for every $\xi \in \mathbb {S}^{n-1}$ and every Borel set $B \subset \Omega$. By using again [26, Theorem 3.5], we infer that for every $\xi \in \mathbb {S}^{n-1}$ property (3.8) holds true, and also $\hat{u}^\xi _y \in \text {BV}_{loc}(\Omega ^\xi _y)$ for $\mathcal {H}^{n-1}$-a.e. $y \in \Pi ^\xi$. Eventually, condition (3.8) and the specific form of $\lambda$, together with a simple disintegration argument with respect to the projection $\pi _\xi :\mathbb {R}^{n} \rightarrow \Pi ^\xi$, give exactly the validity of (3.7). $\square$

#### Proof of Theorem 3.7

In slicing the jump set we will follow the line developed in [2]. As already explained in the introduction, the main difference is that, in contrast to the case of a generic Riemannian manifold, the flatness of $\mathbb {R}^n$ allows to avoid the use of a Korn-Poincaré type of inequality. We start by introducing a class of relevant measures. Before doing this, we recall a measurability lemma.

##### Lemma 3.9

Let $u :\Omega \rightarrow \mathbb {R}^m$ be $\mathcal {L}^n$-measurable. Then, for every Borel set $B \subset \Omega$ we have that

3.11 $$\begin{aligned}&y \mapsto \sum _{ t \in B^\xi _y } (|[\hat{u}^\xi _y(t)]| \wedge 1)\qquad \text { is }\mathcal {H}^{n-1}\text {-measurable} \end{aligned}$$

3.12 $$\begin{aligned}&\xi \mapsto \int _{\Pi ^\xi } \sum _{ t \in B^\xi _y } \big ( |[\hat{u}^\xi _y(t)]| \wedge 1 \big ) \, \textrm{d}\mathcal {H}^{n-1}(y) \qquad \text { is }\mathcal {H}^{n-1}\text {-measurable}. \end{aligned}$$

##### Proof

See [2, Lemma 4.5]. $$\square $$

Given an $\mathcal {L}^n$-measurable function $u :\Omega \rightarrow \mathbb {R}^n$, by virtue of (3.11) we consider for every $\xi \in \mathbb {S}^{n-1}$ the (outer) Borel regular measure $\eta _\xi$ of $\mathbb {R}^n$ given by

3.13 $$\begin{aligned} \eta _\xi (B)&:= \int _{\Pi ^\xi } \sum _{ t \in B^\xi _y} \big ( |[\hat{u}^\xi _y(t)]| \wedge 1 \big ) \, \textrm{d}\mathcal {H}^{n-1}(y) \qquad B \subset \Omega \text { Borel}\,, \end{aligned}$$

3.14 $$\begin{aligned} \eta _\xi (E)&:= \inf \, \{\eta _\xi (B) : \, E \subset B, \ B \subset \Omega \text { Borel}\}\,. \end{aligned}$$

We note that the definition of $\eta _\xi$ in (3.13) does not depend on the representative of *u* in its Lebesgue class.

##### Definition 3.10

(The outer measure $\mathscr {I}_{u,1}$) Let $u :\Omega \rightarrow \mathbb {R}^n$ be measurable and let $\{\eta _\xi \}_{\xi \in \mathbb {S}^{n-1}}$ be the family of measures in (3.13)–(3.14). By virtue of (3.12), via the classical Caratheodory’s construction, we define the (outer) Borel regular measure $\mathscr {I}_{u,1}$ on $\Omega$ as

$$\begin{aligned} \mathscr {I}_{u,1}(E) := \sup _{\delta >0} \, \inf _{G_\delta } \sum _{B \in G_\delta } \int _{{\mathbb {S}^{n-1}}} \eta _\xi (B) \text {d}\mathcal {H}^{n-1}(\xi ), \end{aligned}$$

whenever $E \subset \Omega$ and where $G_\delta$ is the family of all countable Borel covers of *E* made of sets having diameter less than or equal to $\delta$.

##### Remark 3.11

For every $u \in \text {GBV}^{\mathcal {E}}(\Omega ;\mathbb {R}^n)$ and for every Borel set $B \subset \Omega$, it holds

$$ \mathscr {I}_{u,1}(B)= \int _{{\mathbb {S}^{n-1}}} \eta _\xi (B) \text {d}\mathcal {H}^{n-1}(\xi ) = \int _{{\mathbb {S}^{n-1}}} \Big (\int _{\Pi ^\xi } \sum _{ t \in B^\xi _y} \big ( |[\hat{u}^\xi _y(t)]| \wedge 1 \big ) \, \textrm{d}\mathcal {H}^{n-1}(y) \Big )\text {d}\mathcal {H}^{n-1}(\xi ). $$

Given $E\subset \mathbb {R}^n$, we define the codimension one slices of *E* as

$$ E^\xi _s:=\{y \in \Pi ^\xi +s\xi :y \in E \}. $$

##### Proposition 3.12

Let $\xi \in \mathbb {S}^{n-1}$, $s \in \mathbb {R}$ and let $u\in \text {GBV}^{\mathcal {E}}(\Omega ; \mathbb {R}^n)$. Then for $\mathcal {H}^{n-1}$-a.e. $y \in \Omega _s^\xi$ there exists

3.15 $$\begin{aligned} \mathop {\mathrm {ap-\lim }}\limits _{\begin{array}{c} z \rightarrow y,\\ z \in (H^{\pm }_\xi + s\xi )\cap \Omega \end{array}} u(z)=:u^{\xi \pm }_s(y), \end{aligned}$$

where $H^{+}_\xi :=\{z \in \mathbb {R}^n: z \cdot \xi >0 \}$ and $H^-_\xi$ is defined similarly.

##### Proof

Since $u \in \text {GBV}^{\mathcal {E}}(\Omega ;\mathbb {R}^n)$ we have by definition that

$$ \int _{\mathbb {S}^{n-1}} \hat{\mu }^{\xi }_u(\Omega ) \, d\mathcal {H}^{n-1}(\xi ) = \hat{\mu }_{u,1}(\Omega ) < \infty . $$

Therefore, we can find a basis $\{\xi _1,\ldots ,\xi _n\}$ of $\mathbb {R}^n$ such that $\hat{\mu }_u^{\xi _i}(\Omega )<\infty$ for $i=1,\ldots ,n$. By applying [26, Theorem 5.1], we infer that

$$ \mathop {\mathrm {ap-\lim }}\limits _{\begin{array}{c} z \rightarrow y,\\ z \in (H^{\pm }_{\xi _i} + s\xi _i)\cap \Omega \end{array}} u(z)\cdot \xi _i=:v_{i}^\pm (y) $$

exists for every $i=1,\ldots , n$ and for $\mathcal {H}^{n-1}$-a.e. $y \in \Omega _s^\xi$. Since $\xi _1,\ldots ,\xi _n$ form a basis we can readily conclude that the limit in (3.15) exists and, in particular, $u^{\xi \pm }_s(y)\cdot \xi _i=v_{i}^\pm (y)$. $\square$

Thanks to Proposition 3.12, we are now in a position to identify a precise representative for co-dimension one slices through $(n-1)$-planes.

##### Definition 3.13

Given $\xi \in \mathbb {S}^{n-1}$ and $u:\Omega \rightarrow \mathbb {R}^n$, we define for every $s \in \mathbb {R}$ the function $\bar{u}^\xi _s:\Pi ^\xi +s\xi \rightarrow \Pi ^\xi$ as

$$ \bar{u}^\xi _s (y):=\pi _\xi (u_s^{\xi +}(y)), \quad y \in \Omega ^\xi _s, $$

where $u_s^{\xi +}$ is defined in (3.15).

##### Proposition 3.14

Let $u \in \text {GBV}^{\mathcal {E}}(\Omega ;\mathbb {R}^n)$. Then for $\mathcal {H}^{n-1}$-a.e. $\xi \in \mathbb {S}^{n-1}$ and for $\mathcal {L}^1$-a.e. $s \in \mathbb {R}$ the function $\bar{u}^\xi _s \in \text {GBV}^{\mathcal {E}}(\Omega _s^\xi ;\Pi ^\xi ).$

##### Proof

First of all we notice that the Borel regular measure on $\mathbb {S}^{n-1}$ defined as $B \mapsto \int _{\mathbb {S}^{n-1}} \mathcal {H}^{n-2}(B \cap \Pi ^\xi ) \, d\mathcal {H}^{n-1}(\xi )$ is invariant under the action of the orthogonal group. Hence from [29, Theorem 2.7.7] we infer the existence of a dimensional constant *c*(*n*) such that

3.16 $$\begin{aligned} \mathcal {H}^{n-1}(B)= c(n) \int _{\mathbb {S}^{n-1}} \mathcal {H}^{n-2}(B \cap \Pi ^\xi ) \, d\mathcal {H}^{n-1}(\xi ) \quad \forall B \subset \mathbb {S}^{n-1} \text { Borel set}, \end{aligned}$$

By using (2.3) and (3.16) it holds for $\mathcal {H}^{n-1}$-a.e. $\xi \in \mathbb {S}^{n-1}$

$$\begin{aligned} \infty&>\int _{\Pi ^\xi \cap \mathbb {S}^{n-1}} \hat{\mu }_u^\eta (\Omega ) \text {d}\mathcal {H}^{n-2}(\eta ) \\&=\int _{\Pi ^\xi \cap \mathbb {S}^{n-1}} \Big (\int _{\Pi ^\eta }(\hat{\mu }_u)_y^\eta (\Omega _y^\eta ) \text {d}\mathcal {H}^{n-1}(y)\Big )\text {d}\mathcal {H}^{n-2}(\eta ). \end{aligned}$$

Since , we have

$$\begin{aligned}&\int _{\Pi ^\xi \cap \mathbb {S}^{n-1}} \Big (\int _{\Pi ^\eta }(\hat{\mu }_u)_y^\eta \text {d}\mathcal {H}^{n-1}(y)\Big )\text {d}\mathcal {H}^{n-2}(\eta )\\&= \int _{\Pi ^\xi \cap \mathbb {S}^{n-1}} \Big (\int _{\mathbb {R}} \Big (\int _{\Pi ^\eta \cap (\Pi ^\xi +s\xi )}(\hat{\mu }_u)_y^\eta (\Omega _y^\eta ) \text {d}\mathcal {H}^{n-2}(y)\Big )\text {d}\mathcal {L}^1(s) \Big )\text {d}\mathcal {H}^{n-2}(\eta ). \end{aligned}$$

Finally we observe that, for every $\xi \in \mathbb {S}^{n-1}$ and $\eta \in \Pi ^\xi$, $u \cdot \eta = \pi _\xi (u^+)\cdot \eta$ $\mathcal {L}^n$-a.e., thus we can substitute in the above integral $(\hat{\mu }_u)_y^\eta = (\hat{\mu }_{\bar{u}_s^\xi })_y^\eta$, which concludes the proof. $\square$

Next we prove a key technical proposition.

##### Proposition 3.15

Assume that Theorem 3.7 holds true up to dimension $n-1$. Let $u :\Omega \rightarrow \mathbb {R}^n$ be measurable. Then, it holds

3.17 $$\begin{aligned} \mathscr {I}_{u,1} \big ( \{x \in \Omega :\text {for every } \xi \in \mathbb {S}^{n-1}, x \notin J_{\bar{u}_s^\xi } \text { with } s \text { s.t. } x\in \Pi ^\xi +s\xi \} \big ) = 0\,. \end{aligned}$$

Loosely speaking, condition (3.17) relates the jump sets of the $(n-1)$-dimensional slices of *u* to those of its 1-dimensional slices. More precisely, it states that if a point $x \in \Omega$ is such that all $(n-1)$-dimensional slices of *u* along affine hyperplanes passing through *x* do not exhibit a jump at *x*, then, generically, the same holds for all 1-dimensional slices of *u* along lines passing through *x*. In other words, the measure $\mathscr {I}_{u,1}$, which encodes on average the information on the jump sets of all one-dimensional slices of *u*, concentrates on points *x* for which *at least one* $(n-1)$-dimensional slice of *u* exhibits a jump at *x*.

The proof relies on an application of Fubini’s theorem together with the assumption that the main Theorem 3.7 holds up to dimension $n-1$. However, it is worth emphasizing that the condition “for every $\xi$” in (3.17) *cannot* be relaxed to an almost everywhere condition. Indeed, under our pointwise condition, it follows directly from the definition of *E* that for every $\xi \in \mathbb {S}^{n-1}$

$$ (E \cap (\Pi ^\xi + s\xi )) \cap J_{\hat{u}^\xi _s} = \emptyset \quad \text {for every } s \in \mathbb {R}. $$

By contrast, assuming only an almost everywhere condition would imply, by Fubini’s theorem, that for almost every $\xi \in \mathbb {S}^{n-1}$

$$ (E \cap (\Pi ^\xi + s\xi )) \cap J_{\hat{u}^\xi _s} = \emptyset \quad \text {for } \lambda _\xi \text {-almost every } s \in \mathbb {R}, $$

where $\lambda _\xi$ denotes the projection of $\mathscr {I}_{u,1}$ onto the line $\{ s\xi : \ s \in \mathbb {R} \}$. However, we have no *a priori* information on the measure $\lambda _\xi$; in particular, we do not know whether it is absolutely continuous with respect to the Lebesgue measure $\mathcal {L}^1$ (notice that the very same issue arises when projecting $\mathscr {I}_{u,1}$ on $\Pi ^\xi$). Consequently, the above statement cannot be strengthen to: for almost every $\xi \in \mathbb {S}^{n-1}$

$$ (E \cap (\Pi ^\xi + s\xi )) \cap J_{\hat{u}^\xi _s} = \emptyset \quad \text {for } \mathcal {L}^1\text {-almost every } s \in \mathbb {R}, $$

which is precisely the condition of interest.

By imposing an everywhere condition in the definition of *E*, we will later have to pay the price, specifically in Proposition 3.17, of proving a precise pointwise statement.

##### Proof

Let *E* be the set in (3.17), i.e.,

3.18 $$\begin{aligned} E:=\{x \in \Omega :\text {for every } \xi \in \mathbb {S}^{n-1}, x \notin J_{\bar{u}_s^\xi } \text { with } s \text { s.t. } x\in \Pi ^\xi +s\xi \}. \end{aligned}$$

We show that *E* is $\hat{\mu }_u^1$-measurable. We proceed as in [2, Lemma 4.4]. Let $\tau :=\arctan$, we define $s^{\pm }: \Omega \times \mathbb {S}^{n-1} \times \mathbb {S}^{n-1} \rightarrow \mathbb {R}^n$ and $i^{\pm }: \Omega \times \mathbb {S}^{n-1} \times \mathbb {S}^{n-1} \rightarrow \mathbb {R}^n$ as

$$ s_j^{\pm }(x,\xi , \nu ) := \limsup _{r \searrow 0} \int _{(\Pi ^\xi +(x\cdot \xi ) \xi ) \cap (H_{\nu }^{\pm }+x) \cap B_r(x) } \tau ((\bar{u}^{\xi }_{x \cdot \xi })_j(z)) \, \text {d}\mathcal {H}^{n-1}(z), $$

$$ i_j^{\pm }(x,\xi , \nu ) := \liminf _{r \searrow 0} \int _{(\Pi ^\xi +(x\cdot \xi ) \xi ) \cap (H_{\nu }^{\pm }+x) \cap B_r(x) } \tau ((\bar{u}^{\xi }_{x \cdot \xi })_j(z)) \, \text {d}\mathcal {H}^{n-1}(z), $$

for $j=1,\ldots , n$. We notice that $s^{\pm }$ and $i^{\pm }$ are Borel measurable in $\Omega \times \mathbb {S}^{n-1} \times \mathbb {S}^{n-1}$. Indeed, the integrand functions are Borel measurable in the variables $(x,\xi , \nu , z)$. Hence, Fubini’s theorem implies that the integral functions are Borel measurable in $\Omega \times \mathbb {S}^{n-1} \times \mathbb {S}^{n-1}$. Finally, both liminf and limsup can be computed by restricting $r \in \mathbb {Q}$ because of the continuity of the integrals with respect to *r*. Then, the set

$$ B = \left\{ (x,\xi , \nu ) \in \Omega \times \mathbb {S}^{n-1} \times \mathbb {S}^{n-1} : s^{+}(x,\xi , \nu ) = i^{+}(x,\xi , \nu ), \ s^{-}(x,\xi , \nu ) = i^{-}(x,\xi , \nu ), \right. $$

$$ \left. s^{+}(x,\xi , \nu ) \ne s^{-}(x,\xi , \nu ), \quad s^{\pm }_{j}(x,\xi , \nu ) \in \left( -\frac{\pi }{2}, \frac{\pi }{2} \right) \text { for }j =1, \ldots , n \right\} , $$

is Borel measurable. Furthermore, $\Omega \setminus E = \pi _{1} (B)$, where $\pi _{1} :\Omega \times \mathbb {S}^{n-1} \times \mathbb {S}^{n-1} \rightarrow \Omega$ is the projection over the first component. Then, by the measurable projection Theorem (see for instance [29, Section 2.2.13]) the set $\Omega \setminus E$ is $\hat{\mu }_u^1$-measurable, and the same holds for *E*.

From the very definition of *E*, we note that it holds $(E \cap (\Pi ^\xi +s\xi )) \cap J_{\bar{u}_s^\xi } = \emptyset$ for every $\xi \in \mathbb {S}^{n-1}$ and $s \in \mathbb {R}$. Moreover, we know from Proposition 3.14 that for $\mathcal {H}^{n-1}$-a.e. $\xi \in \mathbb {S}^{n-1}$ and for $\mathcal {L}^1$-a.e $s \in \mathbb {R}$ we also have $\bar{u}_s^\xi \in \text {GBV}^{\mathcal {E}}(\Omega _s^\xi ;\Pi ^\xi )$.

For simplicity let us denote $v:=\bar{u}_s^\xi$. Then, by the assumption that Theorem 3.7 holds true, we have, for $\mathcal {L}^1$-a.e. $s \in \mathbb {R}$,

3.19 $$\begin{aligned} J_{\hat{v}_y^\eta }=(J_{v})_y^\eta \end{aligned}$$

for $\mathcal {H}^{n-2}$-a.e. $\eta \in \mathbb {S}^{n-1}\cap \Pi ^\xi$ and $\mathcal {H}^{n-2}$-a.e. $y \in \Pi ^\eta \cap (\Pi ^\xi +s\xi )$. Since the map $(z,s) \mapsto u^\xi _s(z)$ coincides with the map *u*(*z*) for $\mathcal {H}^{n-1}$-a.e. $z \in \Omega _s^\xi$ and for $\mathcal {L}^1$-a.e. $s \in \mathbb {R}$, and since $\eta \in \Pi ^\xi$, we have as well

$$ u(y+t\eta ) \cdot \eta = \pi _\xi (u^\xi _s(y+t\eta )) \cdot \eta = v(y+t\eta ) \cdot \eta , $$

for $\mathcal {L}^1$-a.e. $s \in \mathbb {R}$, for $\mathcal {H}^{n-2}$-a.e. $y \in \Pi ^\eta \cap (\Pi ^\xi +s\xi )$, and for $\mathcal {L}^1$-a.e. $t \in \Omega ^\eta _y$.

Notice that, by using again that $\eta \in \Pi ^\xi$, we have the equality between sets

3.20 $$\begin{aligned}&\{(s,\eta ,y,t) : \ t \in J_{\hat{v}^\eta _y}, \ s \in \mathbb {R}, \ \eta \in \mathbb {S}^{n-1}\cap \Pi ^\xi , \ y \in \Pi ^\eta \cap (\Pi ^\xi +s\xi ) \} \nonumber \\&= \{(s,\eta ,y,t) : \ t \in J_{{\hat{w}}^\eta _{q}}, \ q=y+s\xi , \ s \in \mathbb {R}, \ \eta \in \mathbb {S}^{n-1}\cap \Pi ^\xi , \ y \in \Pi ^\eta \cap (\Pi ^\xi +s\xi ) \}, \end{aligned}$$

where the Borel measurable function $w :\Omega \rightarrow \mathbb {R}^n$ is defined as $w(z+s\xi ):=u^{\xi +}_s(z)$ (see (3.15)). In particular, the Borel measurability of the set appearing in (3.20) follows from the general fact that the following set

$$ \{ (\theta ,x) \in \mathbb {S}^{n-1} \times \Omega : \ t \in J_{\hat{w}^\theta _q}, \ x=q+t\theta , \ q \in \Pi ^\theta \}, $$

is Borel measurable, whenever $w :\Omega \rightarrow \mathbb {R}^n$ is a Borel measurable map (see [2, Lemma 4.4]). Analogously, one can verify that the set

$$\begin{aligned} \{(s,\eta ,y,t) : \ y+t \eta +s\xi \in (J_v)^\eta _y, \ s,t \in \mathbb {R}, \ \eta \in \mathbb {S}^{n-1}\cap \Pi ^\xi , \ y \in \Pi ^\eta \cap (\Pi ^\xi +s\xi ) \}, \end{aligned}$$

is Borel measurable.

In particular, by virtue of Fubini’s Theorem, from equality (3.19) we infer that for $\mathcal {H}^{n-2}$-a.e. $\eta \in \mathbb {S}^{n-1}\cap \Pi ^\xi$ it holds

$$ J_{\hat{u}_y^\eta } \cap E_y^\eta =J_{\hat{v}_y^\eta } \cap E_y^\eta = (J_v)^\eta _y \cap E^\eta _y = (J_v \cap E)^\eta _y= \emptyset \quad \text {for } \mathcal {H}^{n-1}\text {-a.e. } y \in \Pi ^\eta , $$

where in the last equality we have used the relation $(E \cap (\Pi ^\xi +s\xi )) \cap J_{v} = \emptyset$ valid for every $s \in \mathbb {R}$. Summarizing we have obtained that for $\mathcal {H}^{n-1}$-a.e. $\xi \in \mathbb {S}^{n-1}$

3.21 $$\begin{aligned} J_{\hat{u}_y^\eta } \cap E_y^\eta = \emptyset , \ \ \text { for }\mathcal {H}^{n-2}\text {-a.e. }\eta \in \mathbb {S}^{n-1}\cap \Pi ^\xi \text { and }\mathcal {H}^{n-1}\text {-a.e. }y \in \Pi ^\eta . \end{aligned}$$

Eventually, by (3.16), we infer from (3.21) that

$$ J_{\hat{u}_y^\xi } \cap E_y^\xi = \emptyset , \ \ \text { for }\mathcal {H}^{n-1}\text {-a.e. }\xi \in \mathbb {S}^{n-1}\text { and }\mathcal {H}^{n-1}\text {-a.e. }y \in \Pi ^\xi . $$

From the very definition of the measures $\eta _{\xi }$ (cf. (3.13)) we obtain that $\eta _\xi (E)=0$ for $\mathcal {H}^{n-1}$-a.e. $\xi \in \mathbb {S}^{n-1}$. As a direct consequence of the definition of $\mathscr {I}_{u,1}$ we conclude $\mathscr {I}_{u,1}(E)=0$. $\square$

Before proving the dimensional estimate on the set where the measure $\mathscr {I}_{u,1}$ is concentrated, we need the following proposition.

##### Proposition 3.16

Let $A \subset \mathbb {R}^n$ be measurable. Consider the real vector space $L^0(A)$ made of all Lebesgue equivalence classes of measurable functions $v :A \rightarrow \mathbb {R}$ endowed with the metric $\textrm{d}(\cdot ,\cdot )$ defined as

$$ \textrm{d}(v_1,v_2) := \int _A |v_1-v_2| \wedge 1 \, \textrm{d}x, \ \ v_1,v_2 \in L^0(A), $$

which induces the convergence in measure. Then, any finite dimensional vector subspace $V \subset L^0(A)$ is a complete metric space with respect to the distance $\textrm{d}(\cdot ,\cdot )$.

The proposition above is a direct consequence of Riesz Theorem (see for instance [37, Theorem 1.21]). To keep the presentation self contained, we include a proof in the Appendix A.

Recall the definition of $\Theta ^{*n-1}$ (see [29] for further details)

$$ \Theta ^{*n-1}(\mu ,x) :=\limsup _{r \rightarrow 0} \frac{\mu (B_r(x))}{r^{n-1}}, $$

for every measure $\mu \in \mathcal {M}^+(\Omega )$ and $x \in \Omega$.

##### Proposition 3.17

Let $u \in \textrm{GBV}^{\mathcal {E}}(\Omega ;\mathbb {R}^n)$. Then, for $\hat{\mu }_{u,1}$-a.e. $x \in \Omega$, the condition $\Theta ^{*n-1}(\hat{\mu }_{u,1},x)=0$ implies that $x \notin J_{\bar{u}_{s}^\xi }$ for every $\xi \in \mathbb {S}^{n-1}$, with $s \in \mathbb {R}$ such that $x \in \Pi ^\xi +s\xi$.

The strategy for proving the above proposition is to consider the blow-up $u_r$ of the function *u* at the point *x*. The assumption of vanishing upper $(n-1)$-dimensional density, namely $\Theta ^{*\,n-1}(\hat{\mu }_{u,1},x)=0$, allows us to qualitatively control the asymptotic oscillation of each $(n-1)$-dimensional slice $\overline{u}^\xi$ of *u* at *x* as the scale $r \rightarrow 0$. Through a careful analysis, relying only on basic tools from the fundamental theorem of calculus, we show that the vanishing of the upper $(n-1)$-dimensional density of $\hat{\mu }_{u,1}$ at *x* forces, for every $\xi$, any limit of the rescaled slices $\overline{u}_r^\xi$ to belong to a finite-dimensional vector space made of smooth functions. In particular, such limits cannot display the characteristic jump structure.

##### Proof

In order to simplify the notation we set for every $x \in \Omega$ and $\xi \in \mathbb {S}^{n-1}$

$$\begin{aligned} O^\xi _x(u)&:=|D \hat{u}^\xi _x|(\Omega ^\xi _x \setminus J^1_{\hat{u}^\xi _x}) + \mathcal {H}^0(\Omega ^\xi _x \cap J^1_{ \hat{u}^\xi _x}) \\ O_x(u)&:= \int _{\mathbb {S}^{n-1}}|D \hat{u}^\xi _x|(\Omega ^\xi _x \setminus J^1_{\hat{u}^\xi _x}) + \mathcal {H}^0(\Omega ^\xi _x \cap J^1_{ \hat{u}^\xi _x}) \, \textrm{d}\mathcal {H}^{n-1}(\xi ). \end{aligned}$$

**Step 1:** We claim that

3.22 $$\begin{aligned}&\hat{u}^\xi _x \in \text {BV}_{loc}(\Omega ^\xi _x), \ \ \text { for }(\mathcal {L}^n \otimes \mathcal {H}^{n-1})\text {-a.e. }(x,\xi ) \in \Omega \times \mathbb {S}^{n-1} \end{aligned}$$

3.23 $$\begin{aligned}&\ \ \ \ \ \ (x,\xi ) \mapsto O^\xi _x(u) \ \ \text { is }(\mathcal {L}^n \otimes \mathcal {H}^{n-1})\text {-measurable} \end{aligned}$$

3.24 $$\begin{aligned}&\ \ \ \ \ \ \ \ \ \ \ \ \ \int _{\Omega } O_x(u) \, \text {d}x \le c_n(\Omega ) \hat{\mu }_{u,1}(\Omega ). \end{aligned}$$

Indeed, from the definition of $\text {GBV}^{\mathcal {E}}(\Omega ;\mathbb {R}^n)$, we know that for $\mathcal {H}^{n-1}$-a.e. $\xi \in \mathbb {S}^{n-1}$ we have $\hat{u}^\xi _y \in \text {BV}_{loc}(\Omega ^\xi _y)$ for $\mathcal {H}^{n-1}$-a.e. $y \in \Pi ^\xi$. Equivalently, for $\mathcal {H}^{n-1}$-a.e. $\xi \in \mathbb {S}^{n-1}$ we have $\hat{u}^\xi _{\pi _\xi (x)} \in \text {BV}_{loc}(\Omega ^\xi _{\pi _\xi (x)})$ for $\mathcal {L}^{n}$-a.e. $x \in \Omega$, where $\pi _\xi :\mathbb {R}^n \rightarrow \Pi ^\xi$ denotes the orthogonal projection. Therefore, by using Fubini’s Theorem on the product space $\Omega \times \mathbb {S}^{n-1}$ with measure $\mathcal {L}^n \otimes \mathcal {H}^{n-1}$, and the fact that $\hat{u}^\xi _x= \hat{u}^\xi _{\pi _\xi (x)}$, we immediately infer the validity of (3.22). To prove (3.24), in view of the measurability (3.23) (see Lemma 2.2), it is enough to make use of Fubini’s Theorem and exchange the order of integration in *x* and $\xi$.

**Step 2:** Let us assume by contradiction that the measure $\hat{\mu }_{u,1}$ has null $(n-1)$-density at *x* while the set $\Sigma \subset \mathbb {S}^{n-1}$ defined as

$$ \Sigma :=\{\xi \in \mathbb {S}^{n-1} : x \in J_{\bar{u}^\xi _s}\} $$

is not empty. Fix $\xi \in \Sigma$. By the same arguments used to prove the measurability of the set *E* defined in (3.18), it can be shown that $\Sigma$ is $\mathcal {H}^{n-1}$-measurable.

Let $x \in \Omega$, then $x \in \Pi ^\xi +s\xi$ with $s=x\cdot \xi$. Now define for every $0< r <\text {dist}(x, \partial \Omega )$ the rescaled function $u_{r} :B_1(0) \rightarrow \mathbb {R}^n$ as $u_{r}(z):= u^+(x+rz)$, and $\bar{u}_{r} :B_1(0) \cap \Pi ^\xi \rightarrow \mathbb {R}^n$ as $\bar{u}_r(z):=\pi _\xi (u^+(x+rz))$.

From formula (3.3), we deduce that $u_r \in \text {GBV}^{\mathcal {E}}(B_1(0);\mathbb {R}^n)$ and that $\hat{\mu }^\xi _{u_r}(B_1(0))= r^{1-n} \hat{\mu }^\xi _u(B_r(x))$ for $\mathcal {H}^{n-1}$-a.e. $\xi \in \mathbb {S}^{n-1}$. Let us further denote the map $\phi :\mathbb {R}^n \rightarrow \mathbb {R}^n$ defined as

$$ \phi (z):= {\left\{ \begin{array}{ll} \frac{z}{|z|}, & \text { if } z \ne 0\\ 0, & \text { if } z=0. \end{array}\right. } $$

From our assumption, we have, for $\xi \in \Sigma$, that there exists $\nu \in \Pi ^\xi \cap \mathbb {S}^{n-1}$ such that

3.25 $$\begin{aligned} \bar{u}_{r} \rightarrow f_{\nu ,\xi } \ \text { in measure } \text { as }r \rightarrow 0^+, \ \text { where } \ f_{\nu ,\xi }(z):= a_{\nu ,\xi } \,\text {sign}(z\cdot \nu ) + b_{\nu ,\xi } \end{aligned}$$

for some $a_{\nu ,\xi } \in \mathbb {R}^n \setminus \{0\}$ and $b_{\nu ,\xi } \in \mathbb {R}^n$.

**Step 3:** We claim that there exist a subsequence of radii $r_k \searrow 0$ and vectors $\{z_1,\dotsc ,z_{n2^n}\} \subset B_1(0) \setminus \{0\}$, such that $\{z_{(j-1) n + 1},\ldots , z_{jn}\}$ are linearly independent vectors contained in the *j*-th orthant of $\mathbb {R}^n$, $z_i$ are points of approximate continuity for $u_{r_k}$, while (3.22) holds true for $x=z_i$ and $u=u_{r_k}$, and $O_{z_i}(u_{r_k}) \rightarrow 0^+$ for every $k=1,2,\dotsc$ as $k \rightarrow \infty$. Notice that, once the claim is proved, by the Fundamental Theorem of Calculus together with the concavity of the truncation function, there exists $j=1,\ldots , 2^n$ such that for $\mathcal {H}^{n-1}$-a.e. $z \in B_1(0) \cap \Pi ^\xi$ and for every $i=(j-1)n+1,\dotsc ,jn$, we have $\xi \cdot z_i >0$ and

3.26 $$\begin{aligned} |(u_{r_k}(z_i) - u_{r_k}(z)) \cdot \phi (z-z_i)| \wedge 1&\le |\hat{\mu }_{{u}_r}^{ \phi (z-z_i)}|(B_1(0) ), \ k=1,2,\dotsc \end{aligned}$$

We notice that the right-hand side of (3.26) is $\mathcal {H}^{n-1}$-measurable as a function of *z*. Indeed, we could have chosen the vectors $\{z_1,\dotsc ,z_{n2^n}\}$ so that $\eta \mapsto |\hat{\mu }_{{u}_r}^{ \eta }|(B_1(0) )$ are measurable maps between the $\sigma$-algebra of $\mathcal {H}^{n-1}$-measurable subsets of $\mathbb {S}^{n-1}$ and the Borel $\sigma$-algebra of $\mathbb {R}$ (this follows similarly as in the proof of Lemma 2.2). Then, $z \mapsto |\hat{\mu }_{u_r}^{ \phi (z-z_i)}|(B_1(0) )$ is simply the composition of $\eta \mapsto |\hat{\mu }_{u_r}^{ \eta }| (B_1(0) )$ with $z \mapsto \phi (z-z_i)$, which is Borel measurable and whose preimages of $\mathcal {H}^{n-1}$-negligible sets are $\mathcal {H}^{n-1}$-negligible.

To prove the claim we first notice that the assumption $\Theta ^{n-1}(\hat{\mu }_{u,1},x)=0$ implies that $\hat{\mu }^1_{u_r}(B_1(0)) \rightarrow 0$ as $r \rightarrow 0^+$. By letting $\lambda _r:= \hat{\mu }^1_{u_r}(B_1(0))$ we see from (3.24) applied to $u=u_r$ and $\Omega =B_1(0) \cap \Sigma _j$, where $\Sigma _j$ is the *j*-th orthant, that

$$\begin{aligned} \mathcal {L}^n(\{z \in B_1(0) \cap \Sigma _j : O_{z}(u_r) > c_n(\Omega )\sqrt{\lambda _r} \}) \le \sqrt{\lambda _r}, \ \text { for every }0< r < \text {dist}(x,\partial \Omega ). \end{aligned}$$

Therefore, by passing to a subsequence $r_k \searrow 0$ such that $\sum _k \sqrt{\lambda _{r_k}} \le \mathcal {L}^n(B_1(0))/2^{n+1}$, we have that the set $A:= \bigcap _k \{z \in B_1(0) \cap \Sigma _j: O_{z}(u_{r_k}) \le c_n(\Omega )\sqrt{\lambda _{r_k}} \}$ satisfies $\mathcal {L}^n(A) >0$. In particular, we can find $\{z_{(j-1)n+1},\dotsc ,z_{jn}\} \subset A \cap \Sigma _j$ in generic position such that $z_i$ is an approximate continuity point for $u_{r_k}$ and (3.22) holds true with $u=u_{r_k}$ and $x=z_i$ for every $k=1,2,\dotsc$ and $i=(j-1)n+1,\dotsc ,jn$. The fact that $O_{z_i}(u_{r_k}) \rightarrow 0^+$ for every $i=(j-1)n+1,\dotsc ,jn$ as $k \rightarrow \infty$ is a direct consequence of the fact that $\{z_{(j-1)n+1},\dotsc ,z_{jn}\} \subset A$. The claim is thus proved.

**Step 4:** Fix $j=1, \ldots , 2^n$ such that $\xi \cdot z_{i}>0$ for $i=(j-1)n,\ldots , jn$. For simplicity assume $j=1$. We observe that for every $i = 1, \ldots , n$

3.27 $$\begin{aligned} \lim _{r \rightarrow 0} \hat{\mu }_{u_{r}} ^{\phi ^{+} (z - z_{i})} (B_{1} (0)) = 0 \qquad \text {for }\mathcal {H}^{n-1}\text {-a.e.}~z \in \Pi ^{\xi }\cap B_{1}(0) \end{aligned}$$

since $$\Theta ^{*n-1} (\hat{\mu }_{u,1}, x ) = 0$$.

Since $\{z_{1},\dotsc ,z_{n}\}$ are linearly independent vectors, we obtain that

3.28 $$\begin{aligned} \{\phi (z-z_1),\dotsc , \phi (z-z_n)\}\text { is a basis of }\mathbb {R}^n\text { for }\mathcal {H}^{n-1}\text {-a.e. }z \in \Pi ^\xi \cap B_1(0). \end{aligned}$$

Therefore, for $\mathcal {H}^{n-1}$-a.e. $z \in \Pi ^\xi \cap (B_1(0) \setminus B_{1/2}(0))$ we find real smooth coefficients $\{c_1(z),\dotsc ,c_n(z)\}$ such that $\phi (z)=\sum _i c_i(z) \phi (z-z_i)$. In addition, combining (3.26) and (3.28) we infer for every $i=1,\dotsc ,n$ that

$$\begin{aligned} |(u_{r_k}(z_i)-u_{r_k}(z)) \cdot \phi (z-z_i)| \wedge 1\rightarrow 0, \ \text {pointwise for }\mathcal {H}^{n-1}\text {-a.e.}~z \in \Pi ^\xi \cap \big ( B_1(0) \setminus B_{\frac{1}{2}}(0)\big ), \end{aligned}$$

whenever $k \rightarrow \infty$. Therefore, for every sufficiently large *k* (depending on *z*)

$$\begin{aligned}&|u_{r_k}(z) \cdot \phi (z) - \sum _{i} c_i(z)u_{r_k}(z_i) \cdot \phi (z-z_i)| \wedge 1 \\&\le \sum _i |c_i(z)| |(u_{r_k}(z)-u_{r_k}(z_i))\cdot \phi (z-z_i)| \wedge 1\\&\le \sum _i |c_i(z)| |\hat{\mu }_{u_r}^{ \phi (z-z_i)}|(B_1(0)) \end{aligned}$$

By (3.27), the above inequality yields that for $\mathcal {H}^{n-1}$-a.e. $z \in \Pi ^{\xi }\cap \big ( B_{1}(0) \setminus B_{\frac{1}{2}}(0)\big )$

$$\begin{aligned} |u_{r_k}(z) \cdot \phi (z) - \sum _{i} c_i(z)u_{r_k}(z_i)\cdot \phi (z-z_i)| \rightarrow 0, \ \text { as }k \rightarrow \infty . \end{aligned}$$

Thus, by recalling (3.25), we have for $\mathcal {H}^{n-1}$-a.e. $z \in \Pi ^{\xi } \cap \big (B_{1}(0) \setminus B_{\frac{1}{2}}(0)\big )$

3.29 $$\begin{aligned} |f_{\nu ,\xi }(z) - \sum _{i} c_i(z)u_{r_k}(z_i) \cdot \phi (z-z_i)| \rightarrow 0, \ \text { as }k \rightarrow \infty . \end{aligned}$$

We conclude by showing that (3.29) gives a contradiction.

Consider the finite-dimensional real vector subspace *V* of $L^0\big (\Pi ^{\xi } \cap \big (B_{1}(0) \setminus B_{\frac{1}{2}} (0)\big ); \mathbb {R}^{n}\big )$ generated by the elements $\{v_{ij}:\Pi ^{\xi } \cap \big (B_{1}(0) \setminus B_{\frac{1}{2}} (0) \big ) \rightarrow \mathbb {R}^{n}: i,j =1,\dotsc ,n\}$, where $v_{ij}(z):= c_i(z) \phi _j (z-z_i)$ ($\phi _j:= \phi \cdot e_j$). Condition (3.29) combined with Proposition 3.16 (recall that pointwise convergence implies convergence in measure) implies that the function $f_{\nu ,\xi }$ restricted to $\Pi ^{\xi } \cap \big (B_{1}(0) \setminus B_{\frac{1}{2}} (0) \big )$ belongs to *V*.

Since the generators $v_{ij}$ are all smooth functions, any linear combination of them still belongs to $C^\infty (\Pi ^{\xi } \cap \big (B_{1}(0) \setminus B_{\frac{1}{2}} (0)\big ); \mathbb {R}^{n})$, forcing $a_{\nu ,\xi }=0$. This is not possible because we assumed at the beginning $a_{\nu , \xi } \ne 0$ and we reach a contradiction. The proof is thus concluded. $\square$

We are now in position to prove Theorem 3.7.

##### Proof of Theorem 3.7

We proceed by induction. Observe that in dimension one the theorem is trivially true. Now, assume that the theorem holds true in dimension $n-1$. Therefore, from Proposition 3.15 we infer that the measure $\mathscr {I}_{u,1}$ concentrates on the complement in $\Omega$ of the set *E* defined in (3.18). Since by Proposition 3.17 we have that $\Omega \setminus E \subset \{x \in \Omega : \Theta ^{*n-1}(\hat{\mu }_{u,1},x) >0\}$, then by applying the classical density estimates for Radon measure (cf. [29]) we infer that $\Omega \setminus E$ is $\sigma$-finite with respect to $\mathcal {H}^{n-1}$. We are thus in position to apply Besicovitch-Federer structure theorem [29] to write $\Omega \setminus E = R \cup U$ where $R \subset \Omega$ is countably $(n-1)$-rectifiable while the Borel set $U \subset \Omega$ satisfies

$$ \mathcal {H}^{n-1}(\pi _\xi (U)) =0, \ \ \text { for }\mathcal {H}^{n-1}\text {-a.e. }\xi \in \mathbb {S}^{n-1}. $$

Eventually, from the very construction of $\mathscr {I}_{u,1}$, the above property immediately implies that $\mathscr {I}_{u,1}(U)=0$ and hence $\mathscr {I}_{u,1}$ concentrates on the rectifiable set *R*. In particular we deduce the fundamental inclusion

$$\begin{aligned} J_{\hat{u}^\xi _y} \subset R^\xi _y, \ \ \text { for }\mathcal {H}^{n-1}\text {-a.e. }\xi \in \mathbb {S}^{n-1}\text { and for }\mathcal {H}^{n-1}\text {-a.e. }y \in \Pi ^\xi . \end{aligned}$$

We can then conclude by arguing as in the proof of [2, Theorem 4.12]. $\square$

#### Proof of Theorem 3.8

The proof follows the line of [26, Theorem 9.1], we report here only the main differences.

We set

3.30 $$\begin{aligned} \Xi :=\{\xi \in \mathbb {R}^n \setminus \{0\}: \hat{u}_y^\xi \in \text {BV}(\Omega _y^\xi ) \text { for a.e. } y \in \Pi ^\xi \}. \end{aligned}$$

Observe that $\mathcal {L}^n(\mathbb {R}^n \setminus \Xi )=0$. Without loss of generality, we may assume that *u* is a Borel function with compact support in $\Omega$ and that $\hat{u}_y^\xi \in \text {BV}(\Omega _y^\xi )$ for every $\xi \in \Xi$ and for every $y \in \Pi ^\xi$. For every $x \in \Omega$ we define

3.31 $$\begin{aligned} \hat{u}^\xi (x) :=\limsup _{\rho \rightarrow 0^+}\frac{1}{2\rho }\int _{-\rho }^\rho u(x+s\xi )\cdot \xi \, \text {d}s, \end{aligned}$$

$$\begin{aligned} e^\xi (x) :=\limsup _{\rho \rightarrow 0^+}\frac{1}{2\rho }\int _{0}^\rho \frac{\hat{u}^\xi (x+s\xi )-\hat{u}^\xi (x)}{s} \,\text {d}s. \end{aligned}$$

We observe that $u^\xi$ and $e^\xi$ are Borel functions and have compact support on $\Omega$. In particular,

3.32 $$\begin{aligned} e^{\rho \xi }(x)=\rho ^2 e^\xi (x) \quad \text {for every } \rho >0 \text { and every } x \in \Omega . \end{aligned}$$

By the Lebesgue Differentation Theorem, for every $y \in \Pi ^\xi$ we have

$$\begin{aligned} (\hat{u}^\xi )_y^\xi =\hat{u}^\xi _y \quad \mathcal {L}^1\text {-a.e. in } \Omega _y^\xi . \end{aligned}$$

Since $\hat{u}_y^\xi \in \text {BV}(\Omega _y^\xi )$ and $(\hat{u}^\xi )_y^\xi$ is a good representative of $\hat{u}_y^\xi$ by (3.31), we obtain that

3.33 $$\begin{aligned} \nabla \hat{u}_y^\xi (t)=\lim _{s \rightarrow 0} \frac{(\hat{u}^\xi )_y^\xi (t+s)-(\hat{u}^\xi )_y^\xi (t)}{s}=(e^\xi )_y^\xi (t) \end{aligned}$$

for every $y \in \Pi ^\xi$ and for $\mathcal {L}^1$-a.e. $t \in \Omega _y^\xi$.

As in [26, Theorem 9.1], the following parallelogram identity holds

3.34 $$\begin{aligned} e^{\xi +\eta }(x)+e^{\xi -\eta }(x)=2e^\xi (x)+2e^\xi (x) \quad \text {for a.e. } x \in \Omega , \end{aligned}$$

for every $\xi , \eta \in \mathbb {R}^n$ such that $\xi , \eta , \xi +\eta , \xi -\eta \in \Xi$.

Recall (3.30). Let $\xi _1 \in \Xi$. By induction, we consider

$$\begin{aligned} \xi _k \in \Xi _k:=\Xi \cap \Bigg (\bigcap _{1\le i \le k-1 } \bigcap _{q \in \mathbb {Q}}\Xi +q\xi _i \Bigg ) \end{aligned}$$

and we remark that $\mathcal {L}^n(\mathbb {R}^n \setminus \Xi _k)=0$. Define *X* as the vector space over $\mathbb {Q}$ generated by $\{\xi _k\}_{k \in \mathbb {N}}$. Since $\mathcal {L}^n(\mathbb {R}^n \setminus \Xi _k)=0$ for every $k \in \mathbb {N}$, the sequence $\{\xi _k\}_{k \in \mathbb {N}}$ can be chosen to be dense in $\mathbb {R}^n$. We remark that, since $\Xi$ is closed by multiplication with scalars, then by construction $X \subset \Xi$. Since *X* is countable and owing to (3.34), there exists a Borel set $N \subset \Omega$ such that $\mathcal {L}^n(N)=0$ and the parallelogram identity

3.35 $$\begin{aligned} e^{\xi +\eta }(x)+e^{\xi -\eta }(x)=2e^\xi (x)+2e^\xi (x) \end{aligned}$$

holds for every $x \in \Omega \setminus N$ and for every $\xi , \eta \in X$.

Since $e^\xi (x)$ is also positively homogeneous of degree 2 by (3.32), we deduce by [25, Proposition 11.9] that for every $x \in \Omega \setminus N$ there exists a symmetric bilinear form $B_x:X\times X \rightarrow \mathbb {R}$ such that

$$\begin{aligned} e^\xi (x)=B_x(\xi ,\xi ) \end{aligned}$$

for every $\xi \in X$. This implies that for every $x\in \Omega \setminus N$ there exists a symmetric matrix $e(u)(x)\in \mathbb {M}^{n \times n}_{sym}$ such that

3.36 $$\begin{aligned} e^\xi (x)=e(u)(x) \xi \cdot \xi \end{aligned}$$

for every $$\xi \in X$$.

Let us fix $\xi _0 \in \Xi$. We want to prove that (3.36) holds for $\xi = \xi _0$ and for a.e. $x \in \Omega$. Let $X_0$ be the vector subspace over $\mathbb {Q}$ generated by $X \cup \{\xi _0\}$. Since $X_0$ is countable, there exists a Borel set $N_0 \subset \Omega$, with $N \subset N_0$ and $\mathcal {L}^n(N_0)=0$, such that (3.35) holds for every $x \in \Omega \setminus N_0$ and for every $\xi , \eta \in X_0$. Arguing as before, we prove that for every $x \in \Omega \setminus N_0$ there exists a symmetric matrix $A(x) \in \mathbb {M}^{n \times n}_{sym}$ such that

3.37 $$\begin{aligned} e^\xi (x)=A(x)\xi \cdot \xi \end{aligned}$$

for every $\xi \in X_0$. Since $X \subset X_0$ and $N \subset N_0$, equalities (3.36) and (3.37) hold for every $x \in \Omega \setminus N_0$ and for every $\xi \in X$. This implies that $A(x)=e(u)(x)$ for every $x \in \Omega \setminus N_0$. Since (3.37) holds for every $x\in \Omega \setminus N_0$ and for every $\xi \in X_0$, we deduce that the same is true for (3.36). Since $\xi _0 \in X_0$, we conclude that (3.36) holds for $\xi =\xi _0$ for every $x \in \Omega \setminus N_0$.

By the arbitrariness of $\xi _0$ we have shown that for a.e. $\xi \in \mathbb {R}^n$ we have

$$\begin{aligned} e^\xi (x)=e(u)(x)\xi \cdot \xi \quad \text {a.e. in } \Omega . \end{aligned}$$

Lastly, (3.6) follows by combining the above equality and (3.33).

Finally, the fact that $e(u) \in L^1(\Omega ;\mathbb {M}^{n\times n}_{sym})$ follows from

$$\begin{aligned} \begin{aligned} \int _{\Omega }|e(u)(x)| \,\text {d}x&\le c(n) \int _{\Omega }\int _{\mathbb {S}^{n-1}} |e(u)(x)\xi \cdot \xi | \,\text {d}\xi \, \text {d}x\\&= \int _{\mathbb {S}^{n-1}} \int _{\Pi ^\xi } \int _{\Omega _y^\xi } |e(u)_y^\xi (t)\xi \cdot \xi )| \,\text {d}t \,\text {d}y \, \text {d}\xi \\&\le \hat{\mu }_{u,1}(\Omega ) <\infty . \end{aligned} \end{aligned}$$

## Proof of Theorem 1.1

We divide this section into two subsections. The first one contains a number of preliminary results that will be exploited in the second subsection, where the proof of Theorem 1.1 is carried out.

### Preliminary results

We start with a general estimate.

#### Lemma 4.1

For every positive integer *n* it holds true

4.1 $$\begin{aligned} \sup _{e \in \mathbb {R}^{n}} \int _{B_4(0)\setminus B_{1/4}(0)} \frac{|e|}{1+|e \cdot \eta |^2} \, \text {d}\mathcal {H}^n(\eta ) < \infty . \end{aligned}$$

#### Proof

An application of Coarea Formula with the map $f :\mathbb {R}^n \rightarrow \mathbb {R}$ defined as $f(\eta ):= |\eta |$ allows us to write

$$\begin{aligned} \sup _{e \in \mathbb {R}^n}&\int _{B_4(0)\setminus B_{1/4}(0)} \frac{|e|}{1+|e \cdot \eta |^2} \, \text {d}\mathcal {H}^n(\eta ) \\&=\sup _{e \in \mathbb {R}^n} \int _{1/4}^4 \bigg ( \int _{\partial B_\rho (0)} \frac{|e|}{1+|e \cdot \eta |^2} \, \textrm{d}\mathcal {H}^{n-1}(\eta ) \bigg ) \,\textrm{d}\rho \\&= \sup _{e \in \mathbb {R}^n}\int _{1/4}^4 \bigg ( \int _{\partial B_1(0)} \frac{\rho ^{n-1}|e|}{1+\rho ^2|e \cdot \eta |^2} \, \textrm{d}\mathcal {H}^{n-1}(\eta ) \bigg )\, \textrm{d}\rho \\&\le 4^n \sup _{e \in \mathbb {R}^n}\int _{\mathbb {S}^{n-1}} \frac{|e|}{1+|e \cdot \eta |^2} \, \textrm{d}\mathcal {H}^{n-1}(\eta ) . \end{aligned}$$

Hence, it is enough to prove

4.2 $$\begin{aligned} \sup _{e \in \mathbb {R}^n} \int _{\mathbb {S}^{n-1}} \frac{|e|}{1+|e \cdot \eta |^2} \, \text {d}\mathcal {H}^{n-1}(\eta ) <\infty . \end{aligned}$$

We remark that, since the integrand in (4.2) is rotation invariant, we can assume $e=\lambda e_1$, where $\lambda \in \mathbb {R}$ and $e_1$ is the first element of the canonical basis. Moreover, we observe that by setting $\eta _1:= \eta \cdot e_1$ it is enough to show

$$\begin{aligned} \sup _{\lambda >0}\int _{\mathbb {S}_\delta ^{n-1}} \frac{\lambda }{1+\lambda ^2\eta _1^2} \, \text {d}\mathcal {H}^{n-1} (\eta ) < \infty \end{aligned}$$

for some $\delta \in (0,1)$, where $\mathbb {S}_\delta ^{n-1}=\mathbb {S}^{n-1}\cap \{0\le \eta _1\le \delta \}.$ Indeed, the integral

$$\begin{aligned} \int _{\mathbb {S}^{n-1} \cap \{\eta _1>\delta \}} \frac{\lambda }{1+\lambda ^2\eta _1^2} \, \text {d}\mathcal {H}^{n-1} (\eta ) \end{aligned}$$

is uniformly bounded by a constant only depending on *n* and $\delta$.

By applying the Coarea Formula with the map $f :\mathbb {S}^{n-1} \rightarrow \mathbb {R}$ defined as $f(\eta ):= \eta _1$ we have

$$\begin{aligned} \int _{\mathbb {S}_\delta ^{n-1}}&\frac{\lambda }{1+\lambda ^2\eta _1^2} \frac{|\text {J}_{\mathbb {S}^{n-1}}f(\eta )|}{|\text {J}_{\mathbb {S}^{n-1}}f(\eta )|} \, \text {d}\mathcal {H}^{n-1} (\eta ) \\&\le \sup _{\eta \in \mathbb {S}_\delta ^{n-1}} \frac{1}{|\text {J}_{\mathbb {S}^{n-1}}f(\eta )|} \int _0^1 \frac{\lambda }{1+\lambda ^2t^2}\mathcal {H}^{n-2}(\mathbb {S}_\delta ^{n-1} \cap \{\eta _1=t\}) \, \text {d}t\\&\le C(n,\delta ) \int _0^1 \frac{\lambda }{1+\lambda ^2t^2} \, \text {d}t=C(n,\delta )\arctan (\lambda ) \le C(n,\delta )\frac{\pi }{2}. \end{aligned}$$

This concludes the proof of (4.1). $\square$

We now recall some useful results shown in [31] for the study of nonlocal approximations of the Mumford-Shah functional. For the reader’s convenience, we report the proof of Lemma 4.5, highlighting the ambient space $\Omega$ in the functional. The proofs of Lemmas 4.2-4.4 coincide with those of [31].

#### Lemma 4.2

([31, Lemma 3.2]) Let $I=[a,b]$ be an interval, let $\{u_{\varepsilon }\}_{\varepsilon >0} \subset L^1_{loc}(\mathbb {R})$, and let $u \in L^1_{loc}(\mathbb {R})$. Let us assume the following:

(i) $$u_{\varepsilon } \rightarrow u$$ in $$L^1_{loc}(\mathbb {R});$$
(ii) *a* and *b* are Lebesgue points of *u*.

Then

$$\begin{aligned} \liminf _{\varepsilon \rightarrow 0} F_{\varepsilon }(u_{\varepsilon },I) \ge \min \left\{ \frac{\pi }{2}, \frac{(u(b)-u( a ))^2}{b-a}\right\} . \end{aligned}$$

#### Lemma 4.3

([31, Lemma 3.3]) Let $u \in L^{\infty }(\mathbb {R}).$ Then there exists $a \in \mathbb {R}$ such that

(i) $a+q$ is a Lebesgue point of *u* for every $q \in \mathbb {Q}$;
(ii) every sequence $\{u_k\}_{k \in \mathbb {N}} \subset L^\infty (\mathbb {R})$ that satisfies the following conditions:
  $$u_k(a+\frac{z}{k})=u(a+\frac{z}{k})$$ for all $$z \in \mathbb {Z}$$;
  if $x \in [a+\frac{z}{k},a+\frac{z+1}{k}],$ then $u_k(x)$ belongs to the interval with endpoints $u(a+\frac{z}{k})$ and $u(a+\frac{z+1}{k})$;
  has a subsequence converging to *u* in $L^1_{loc}(\mathbb {R})$.

#### Lemma 4.4

([31, Lemma 5.1]) Let $\Omega \subset \mathbb {R}^n$ be an open set. For every $u\in L^0(\Omega , \mathbb {R}^m)$, every $E \Subset \Omega$, every $\delta >0$, and every $\xi \in \mathbb {R}^n$ such that $E + \delta \xi \subset \Omega$, we have that

$$\begin{aligned} \int _E |\arctan (u(x+\delta \xi ) \cdot \xi )-\arctan (u(x)\cdot \xi )| \text {d}x\le C_E \delta (1+F_{\delta ,\xi }(u, E )), \end{aligned}$$

for some $C_E>0$ only depending on *E*.

#### Lemma 4.5

([31, Lemma 5.3]) Let $\Omega \subset \mathbb {R}^n$ be an open set, $E \Subset \Omega$, $R \subset \mathbb {R}^{n}$, and $\eta >0$ be such that $R\subset \frac{\Omega - \Omega }{\eta }$ and

$$ \textrm{dist} ( E, \partial \Omega ) \ge \eta \, \sup _{\xi \in R} |\xi |\,. $$

Then, for every $u \in L^{0}(\Omega ;\mathbb {R}^n)$, $\varepsilon >0$, and $m \in \mathbb {N}$ such that $m \varepsilon <\eta$ we have that

4.3 $$\begin{aligned} \int _{R} F_{ m \varepsilon , \xi }(u,E ) \, \textrm{d}\xi \le \int _{R} F_{\varepsilon , \xi }(u, \Omega ) \, \textrm{d}\xi \,. \end{aligned}$$

#### Proof

Given $\varepsilon >0$, we prove the statement by induction over *m*. If $m=1$ there is nothing to prove. Let us assume that (4.3) holds for *m* and prove it for $m+1$, assuming that $(m + 1) \varepsilon < \eta$. In particular, this implies that $E \subset \Omega - (m+1) \varepsilon \xi$ for every $\xi \in R$. For $A, B \in \mathbb {R}$ it holds

$$\begin{aligned} \arctan \bigg ( \frac{(A+ B)^{2}}{ m+1 } \bigg ) \le \arctan (A^{2}) + \arctan \bigg ( \frac{B^{2}}{ m } \bigg ) \,. \end{aligned}$$

Applying such inequality to

$$\begin{aligned} A = \frac{ (u(x + ( m+1 ) \varepsilon \xi ) - u(x + m \varepsilon \xi )) \cdot \xi }{ \sqrt{\varepsilon }} \qquad B = \frac{ (u(x + m \varepsilon \xi ) - u(x)) \cdot \xi }{\sqrt{\varepsilon }}\,, \end{aligned}$$

with $x \in E$ and $\xi \in R$, we get that

$$\begin{aligned}&\frac{1}{( m+1 )\varepsilon } \arctan \bigg ( \frac{ \big ( (u(x + (m+1) \varepsilon \xi ) - u(x) ) \cdot \xi \big ) ^{2}}{(m +1) \varepsilon } \bigg ) \\&\quad \le \frac{1}{( m +1) \varepsilon } \arctan \bigg ( \frac{ \big ( ( u ( x + (m+1) \varepsilon \xi ) - u( x + m \varepsilon \xi ) ) \cdot \xi \big )^{2}}{\varepsilon } \bigg ) \\&\qquad + \frac{m}{(m+1)}\frac{1}{ m \varepsilon } \arctan \bigg ( \frac{ \big ( (u(x + m \varepsilon \xi ) - u(x)) \cdot \xi \big )^{2}}{ m \varepsilon } \bigg ) \end{aligned}$$

Integrating over $x \in E$ and $\xi \in R$ and performing a change of variable in the first integral on the right-hand side we obtain

$$\begin{aligned} \int _{R} F_{( m+1 )\varepsilon , \xi } (u, E) \, \textrm{d}\xi&\le \frac{1}{( m+1 ) \varepsilon } \int _{R} \int _{E + m \varepsilon \xi } \arctan \bigg ( \frac{ \big ( ( u ( x + \varepsilon \xi ) - u( x ) ) \cdot \xi \big )^{2}}{\varepsilon } \bigg ) \, \textrm{d}x \, \textrm{d}\xi \\&\qquad + \frac{m}{(m+1)} \int _{R} F_{ m \varepsilon , \xi } (u, E) \, \textrm{d}\xi \\&\le \frac{1}{( m +1)} \int _{R} F_{\varepsilon , \xi } (u, \Omega ) \, \textrm{d}\xi + \frac{m}{(m+1)} \int _{R} F_{ m \varepsilon , \xi } (u, E) \, \textrm{d}\xi \,, \end{aligned}$$

where, in the last inequality, we have used that $E + m \varepsilon \xi \subset \Omega \cap \Omega - \varepsilon \xi$. Hence, we conclude for (4.3) by the induction hypothesis. $\square$

We conclude this section with the following lemma, which characterizes the density $\varphi _{p}$ and the constant $\beta _p$ appearing in (1.7) and in the definition of the functional $\mathcal {F}^{p}$ in (1.10).

#### Lemma 4.6

Let $p \ge 1$, let $\Omega \subset \mathbb {R}^n$ be an open set, and let $\varphi _{p} :\mathbb {M}^{n \times n}_{sym} \rightarrow [0,\infty )$ be the map with quadratic growth defined as

$$\begin{aligned} \varphi _{p} (A)&:= \bigg (\int _{\mathbb {R}^n} \left( |A \xi \cdot \xi |^2 \right) ^{p} e^{-|\xi |^2} \text {d}\xi \bigg )^{\frac{1}{p}} \qquad \text {for }A \in \mathbb {M}^{n \times n}_{sym}\text { and }p <\infty . \end{aligned}$$

Then, there exists a positive constant $\beta _{p} >0$ such that for every $u \in \text {GSBD}(\Omega ;\mathbb {R}^n)$ it holds

$$\begin{aligned} \sup _{\mathscr {B}} \sum _{B \in \mathscr {B}}\frac{\pi }{2}&\left( \int _{\mathbb {R}^n} \left( \int _{\Pi ^\xi } \mathcal{M}\mathcal{S}_{\frac{2}{\pi }}(\hat{u}_y^\xi ,B_y^\xi ) \, \text {d}\mathcal {H}^{n-1}(y) \right) ^{p}|\xi |^p e^{-|\xi |^2} \text {d}\xi \right) ^{\frac{1}{p}}\\&=\!\int _{\Omega } \varphi _{p} (e(u))\, \text {d}x + \beta _p \mathcal {H}^{n-1}(J_{u}), \end{aligned}$$

where $\mathscr {B}$ is the set of all possible finite families of pairwise disjoint balls in $\Omega$.

#### Proof

Let $\xi \in \mathbb {R}^n$. We recall that

$$\begin{aligned} \frac{\pi }{2}\mathcal{M}\mathcal{S}_{\frac{2}{\pi }}(\hat{u}_y^\xi ,B_y^\xi )= \int _{B_y^\xi }|\nabla \hat{u}_y^\xi |^2 \text {d}\tau + \frac{\pi }{2}\mathcal {H}^{0}(J_{\hat{u}_y^\xi } \cap B_y^\xi ) . \end{aligned}$$

Integrating the expression above in $y \in \Pi ^\xi$, by Theorem 3.8 we obtain for the first term

$$\begin{aligned} \begin{aligned} \int _{\Pi ^\xi } \int _{B_y^\xi } |\xi | | \nabla \hat{u}^{\xi }_{y}|^{2}\, \textrm{d}\tau \, \textrm{d}\mathcal {H}^{n-1} (y)&= \int _{\Pi ^\xi } \int _{B_y^\xi } |\xi ||\nabla _{\tau } u(y+\tau \xi )\cdot \xi |^2 \, \text {d}\tau \, \text {d}\mathcal {H}^{n-1}(y) \\&=\int _{\Pi ^\xi } \int _{B_y^\xi } |\xi ||e(u)(y+\tau \xi )\xi \cdot \xi |^2 \, \text {d}\tau \, \text {d}\mathcal {H}^{n-1}(y)\\&=\int _{B} |e(u)(x )\xi \cdot \xi |^2 \, \text {d}x. \end{aligned} \end{aligned}$$

On the other hand, by the Area Formula and Theorem 3.7, for the second term we get

$$\begin{aligned} \begin{aligned} \int _{\Pi ^\xi } |\xi |\mathcal {H}^0(J_{\hat{u}^\xi _y} \cap B)\, \text {d}\mathcal {H}^{n-1}(y)= \int _{J_{u} \cap B} |\xi | |\nu _u(x)\cdot \xi |\, \text {d}\mathcal {H}^{n-1}(x). \end{aligned} \end{aligned}$$

We define for every ball $B \subset \Omega$ the function

$$\begin{aligned} \zeta (u,B):=\left( \int _{\mathbb {R}^n} \left( \int _{B} |e(u)(x) \xi \cdot \xi |^2 \text {d}x +\frac{\pi }{2}|\xi |\int _{J_u\cap B} |\nu _u(x) \cdot \xi |\, \text {d}\mathcal {H}^{n-1}(x) \right) ^{p} e^{-|\xi |^2} \, \text {d}\xi \right) ^{\frac{1}{p}}, \end{aligned}$$

and for every open $A \subset \Omega$ we set

$$\begin{aligned} \mu (u,A):=\sup _{\mathscr {B}_A} \sum _{B \in \mathscr {B}_A} \zeta (u,B), \end{aligned}$$

where $\mathscr {B}_A$ is any finite family of disjoint balls contained in *A*. Then, $\mu$ can be extended to a Borel measure (which we still denote by $\mu$ with a slight abuse of notation). We also observe that there exists a positive constant *C* only depending on *n* and *p* such that for every open set $A \subset \Omega$ we have

4.4 $$\begin{aligned} \mu (u,A) \le C \mathcal {G}_{\frac{2}{\pi }}(u,A), \end{aligned}$$

where the inequality follows by the definition of $\mu$. In particular, (4.4) holds for every Borel set. Hence, we decompose $\mu$ in the following way

$$\begin{aligned} \mu (u,B)= \int _{B} f(x)\, \text {d}x + \int _{J_u \cap B} g(x) \, \text {d}\mathcal {H}^{n-1}(x) \end{aligned}$$

for every Borel set $B \subset \Omega$, for some densities *f* and *g*.

We start by calculating the density *f*, which is given by

$$\begin{aligned} f(x)=\lim _{r \rightarrow 0} \frac{\mu (u,B_r(x))}{\omega _n r^n} \end{aligned}$$

for $\mathcal {L}^n$-a.e. $x \in \Omega$. Let $x \in \Omega$ be such that

4.5 $$\begin{aligned}&\lim _{r \rightarrow 0}\frac{1}{r^n}\int _{B_r(x)} |e(u)(y)-e(u)(x)|^2 \text {d}y =0, \end{aligned}$$

4.6 $$\begin{aligned}&\lim _{r \rightarrow 0}\frac{\mathcal {H}^{n-1}(J_u \cap B_r(x))}{r^n}=0\,. \end{aligned}$$

We recall that $\mathcal {L}^n$-a.e. $x \in \Omega$ satisfy (4.5) and (4.6). Consider $\mathscr {B}_{B_r(x)}$ a family of covers of $B_r(x)$ such that

4.7 $$\begin{aligned} \lim _{r \rightarrow 0}&\frac{\mu (u,B_r(x)) -\sum _{B \in \mathscr {B}_{B_r(x)}} \zeta (u,B)}{r^{n}} = 0 \,,\end{aligned}$$

4.8 $$\begin{aligned} \lim _{r\rightarrow 0}&\frac{\mathcal {L}^n(B_r(x))- \sum _{B \in \mathscr {B}_{B_r(x)}}\mathcal {L}^n ( B ) }{r^{n}} = 0 \,. \end{aligned}$$

In view of (4.7), for every $\delta >0$ we have

4.9 $$\begin{aligned} f(x)&= \lim _{r \rightarrow 0} \frac{\mu (u,B_r(x))}{\omega _n r^n} = \lim _{r \rightarrow 0} \frac{\sum _{B \in \mathscr {B}_{B_r(x)}} \zeta (u,B)}{\omega _n r^n} \nonumber \\&=\lim _{r \rightarrow 0} \frac{1}{\omega _n r^n}\sum _{B \in \mathscr {B}_{B_r(x)}} \Bigg (\int _{\mathbb {R}^n} \Big ( \int _B( |(e(u)(y)-e(u)(x)+e(u)(x))\xi \cdot \xi |^2 \text {d}y \nonumber \\&\quad +\frac{\pi }{2}|\xi |\int _{J_u\cap B} |\nu _u(y) \cdot \xi |\, \text {d}\mathcal {H}^{n-1}(y) \Big )^{p} e^{-|\xi |^2} \text {d}\xi \Bigg )^{\frac{1}{p}} \nonumber \\&\le \lim _{r \rightarrow 0} \frac{1}{\omega _n r^n}\sum _{B \in \mathscr {B}_{B_r(x)}} \Big (\int _{\mathbb {R}^n} (1+\delta )^p |e(u)(x) \xi \cdot \xi |^{2p} \mathcal {L}^n(B)^p e^{-|\xi |^2} \text {d}\xi \Big )^{\frac{1}{p}}\end{aligned}$$

4.10 $$\begin{aligned}&\quad + \lim _{r \rightarrow 0} \frac{1}{\omega _n r^n}\sum _{B \in \mathscr {B}_{B_r(x)}}\bigg (\int _{\mathbb {R}^n} \Big (\int _B \big (1+\frac{1}{\delta }\big ) |e(u)(y)-e(u)(x)|^2 \text {d}y \Big )^p |\xi |^{4p} e^{-|\xi |^2} \text {d}\xi \bigg )^{\frac{1}{p}} \end{aligned}$$

4.11 $$\begin{aligned}&\quad +\lim _{r \rightarrow 0} \frac{1}{\omega _n r^n}\sum _{B \in \mathscr {B}_{B_r(x)}}\bigg (\int _{\mathbb {R}^n}\Big (\frac{\pi }{2}\int _{J_u\cap B} |\nu _u(y) \cdot \xi |\, \text {d}\mathcal {H}^{n-1}(y) \Big )^{p} |\xi |^p e^{-|\xi |^2} \text {d}\xi \bigg )^{\frac{1}{p}}. \end{aligned}$$

The limit in (4.10) rewrites as

$$\begin{aligned} \big (1+\frac{1}{\delta }\big ) \bigg (\int _{\mathbb {R}^n} |\xi |^{4p} e^{-|\xi |^2} \text {d}\xi \bigg )^{\frac{1}{p}} \lim _{r \rightarrow 0} \frac{1}{\omega _n r^n} \sum _{B \in \mathscr {B}_{B_r(x)}} \int _B |e(u)(y)-e(u)(x)|^2 \text {d}y \end{aligned}$$

which is equal to zero by (4.5). The limit in (4.11) is bounded by

$$\begin{aligned} \frac{\pi }{2} \bigg (\int _{\mathbb {R}^n} |\xi |^{2p} e^{-|\xi |^2} \text {d}\xi \bigg )^{\frac{1}{p}} \lim _{r \rightarrow 0} \frac{1}{\omega _n r^n}\sum _{B \in \mathscr {B}_{B_r(x)}} \mathcal {H}^{n-1}(J_u \cap B) \end{aligned}$$

which is also equal to zero by (4.6). Finally, by (4.8) the limit in (4.9) is equal to

$$\begin{aligned} (1+\delta )\left( \int _{\mathbb {R}^n} \left( |e(u)(x) \xi \cdot \xi |^2 \right) ^{p} e^{-|\xi |^2} \text {d}\xi \right) ^{\frac{1}{p}}= (1+\delta )\varphi _{p} \big (e(u)(x)\big ) \end{aligned}$$

for every $\delta >0$, and thus $f(x)\le \varphi _{p} \big (e(u)(x)\big )$. The other inequality follows from similar arguments.

In order to calculate the density *g* we consider $x \in J_u$ such that

4.12 $$\begin{aligned}&\lim _{r \rightarrow 0}\frac{\int _{B_r(x)}|e(u)(y)\xi \cdot \xi |^2\text {d}y}{r^{n-1}} =0,\end{aligned}$$

4.13 $$\begin{aligned}&\lim _{r \rightarrow 0} \frac{\mathcal {H}^{n-1}(J_u \cap B_r(x))}{\omega _{n-1} r^{n-1}} =1,\end{aligned}$$

4.14 $$\begin{aligned}&\lim _{r \rightarrow 0}\frac{ 1}{\omega _{n-1} r^{n-1}} \int _{B_r(x) \cap J_u} |\nu _u(y) -\nu _u(x)| \, \text {d}\mathcal {H}^{n-1}(y) = 0. \end{aligned}$$

We remark that $\mathcal {H}^{n-1}$-a.e. $x \in J_u$ satisfy (4.12)–(4.14). We consider a family of covers $\mathscr {B}_{B_r(x)}$ of $B_r(x)$ such that

4.15 $$\begin{aligned} \lim _{r\rightarrow 0}&\frac{ \mu (u,B_r(x)) - \sum _{B \in \mathscr {B}_{B_r(x)}} \zeta (u,B)}{r^{n-1}} = 0\,. \end{aligned}$$

Thanks to (4.15) we obtain

4.16 $$\begin{aligned} g(x)&= \lim _{r \rightarrow 0} \frac{\mu (u,B_r(x))}{\omega _{n-1} r^{n-1}} = \lim _{r \rightarrow 0} \frac{\sum _{B \in \mathscr {B}_{B_r(x)}} \zeta (u,B)}{\omega _{n-1} r^{n-1}} \nonumber \\&=\lim _{r \rightarrow 0} \frac{1}{\omega _{n-1} r^{n-1}}\hspace{-0.05cm}\sum _{B \in \mathscr {B}_{B_r(x)}} \hspace{-0.05cm}\bigg (\int _{\mathbb {R}^n} \Big ( \frac{\pi }{2}|\xi |\int _{J_u\cap B} |(\nu _u(y)-\nu _u(x) +\nu _u(x)) \cdot \xi |\, \text {d}\mathcal {H}^{n-1}(y) \nonumber \\&\quad + \int _B( |e(u)(y)\xi \cdot \xi |^2 \text {d}y \Big )^{p} e^{-|\xi |^2} \text {d}\xi \bigg )^{\frac{1}{p}} \nonumber \\&\le \lim _{r \rightarrow 0} \frac{1}{\omega _{n-1} r^{n-1}}\hspace{-0.05cm}\sum _{B \in \mathscr {B}_{B_r(x)}}\hspace{-0.05cm}\bigg (\int _{\mathbb {R}^n}\Big (\frac{\pi }{2}\int _{J_u\cap B} |\nu _u(x) \cdot \xi |\, \text {d}\mathcal {H}^{n-1}(y) \Big )^{p} |\xi |^p e^{-|\xi |^2} \text {d}\xi \bigg )^{\frac{1}{p}} \end{aligned}$$

4.17 $$\begin{aligned}&\quad +\lim _{r \rightarrow 0} \frac{1}{\omega _{n-1} r^{n-1}}\hspace{-0.1cm}\sum _{B \in \mathscr {B}_{B_r(x)}}\hspace{-0.1cm}\bigg (\int _{\mathbb {R}^n}\Big (\frac{\pi }{2}\int _{J_u\cap B} |\nu _u(y)-\nu _u(x) |\, \text {d}\mathcal {H}^{n-1}(y) \Big )^{p} |\xi |^{2p} e^{-|\xi |^2} \text {d}\xi \bigg )^{\frac{1}{p}}\end{aligned}$$

4.18 $$\begin{aligned}&\quad + \lim _{r \rightarrow 0} \frac{1}{\omega _{n-1} r^{n-1}}\sum _{B \in \mathscr {B}_{B_r(x)}}\bigg (\int _{\mathbb {R}^n} \Big (\int _B |e(u)(y)|^2 \text {d}y \Big )^p |\xi |^{4p} e^{-|\xi |^2} \text {d}\xi \bigg )^{\frac{1}{p}}. \end{aligned}$$

Similarly as before, the limit in (4.17) is equal to zero by (4.13) and (4.14). From (4.12) it follows that also (4.18) is zero. Finally, we observe that (4.16) is equal to

$$\begin{aligned} \frac{\pi }{2} \left( \int _{\mathbb {R}^n} |\nu _u(x) \cdot \xi |^{p}|\xi |^p e^{-|\xi |^2} \text {d}\xi \right) ^{\frac{1}{p}}=:\beta _{p}, \end{aligned}$$

hence $g(x) \le \beta _p$. The reverse inequality follows from similar computations. $\square$

#### Remark 4.7

We observe that, in the case $p=1$, the function $\varphi _1$ admits a more explicit representation: for every $A \in \mathbb {M}^{n\times n}_{sym}$ we have

4.19 $$\begin{aligned} \varphi _1(A)=\int _{\mathbb {R}^n} \langle A \xi , \xi \rangle ^2 e^{-|\xi |^2} \text {d}\xi = \frac{\pi ^{n/2}}{4} (2|A|^2+\text {tr}(A)^2). \end{aligned}$$

To show it we proceed by induction on the dimension *n*. We first observe that, without loss of generality, we can assume the matrix *A* to be diagonal, $a_i:=A_{ii}$. If $n=1$, by a simple calculation we get

$$\begin{aligned} \int _{\mathbb {R}}a^2 \xi ^4 e^{-\xi ^2} \text {d}\xi = \frac{\pi ^{1/2}}{4} 3a^2. \end{aligned}$$

Assume now that the thesis holds up to dimension $n-1,$ then we obtain

$$\begin{aligned}&\int _{\mathbb {R}^n} \left( \Big ( \sum _{i=1}^{n-1} a_i \xi _i^2 \Big )^2 + a_n^2 \xi _n^4 + 2 \sum _{i=1}^{n-1} a_i \xi _i^2 a_n \xi _n^2 \right) e^{-|\xi |^2} \text {d}\xi \\&= \frac{\pi ^{n/2}}{4} \big (2 |A|^2_{n-1}+\text {tr}_{n-1}(A)^2\big ) + \frac{\pi ^{n/2}}{4} (2a_n^2+a_n^2) + \frac{\pi ^{n/2}}{4} \sum _{i=1}^{n-1} 2 a_i a_n \\&= \frac{\pi ^{n/2}}{4} \big (2|A|^2+\text {tr}(A)^2\big ), \end{aligned}$$

where by $|A|_{n-1}^2$ we denoted the sum of the square of the first $n-1$ eigenvalues of *A* and, similarly, by $\text {tr}_{n-1}(A)$ we denoted the sum of the first $n-1$ eigenvalues of *A*.

### Proof of Theorem 1.1

Consider any subsequence $\varepsilon _k \rightarrow 0$ and set $u_k:=u_{\varepsilon _k}$. By Hölder’s inequality, we have

$$\begin{aligned} M:= \sup _{k \in \mathbb {N}} \mathcal {F}^1_{\varepsilon _{k}} (u_{k}, \Omega )\le C(n,p) \sup _{k \in \mathbb {N}} \mathcal {F}^p_{\varepsilon _{k}} (u_{k}, \Omega ) <\infty . \end{aligned}$$

We divide the proof into 6 steps. In Steps 1 and 2 we prove that $u_{k}$ converges pointwise, up to an exceptional set *A*, to some limit function *u* by a Fréchet-Kolmogorov argument. Steps 3–6 are devoted to show that *u* is indeed a $\text {GSBV}^{\mathcal {E}}$-function and that *A* is of finite perimeter.

**Step 1:** Fix two open sets $F \Subset E \Subset \Omega$ and define $f_{k}:\Omega \times \mathbb {R}^n \rightarrow \mathbb {R}$ by

$$ f_{k}(x,\xi ):=\tau (u_{k}(x)\cdot \xi ):=\arctan (u_{k}(x)\cdot \xi ). $$

Let $k \in \mathbb {N}$ be sufficiently large so that $B_4(0)\setminus B_{1/4}(0) \subset \frac{\Omega -\Omega }{\varepsilon _k}$ (notice that $\Omega - \Omega$ is an open set containing the origin) and $4\varepsilon _{k} \le \textrm{dist}(E, \partial \Omega )$. In particular, this implies that $F \subset E \subset \Omega -\varepsilon _k t \xi$ for every $\xi \in B_{4}(0)\setminus B_{1/4}(0)$ and every $t \in [0, 1]$. For simplicity of notation, let us set $R:= B_{2}(0) \setminus B_{1/2}(0)$. In order to apply Fréchet-Kolmogorov Theorem on $F \times R$, we start by showing that for every $\alpha >0$ there exist $\bar{t}\in (0,1)$ such that

4.20 $$\begin{aligned} \int _{F \times R} |f_{k}(x+ \eta ,\xi )-f_{k}(x,\xi )|\, \text {d}x \, \text {d}\xi \le \alpha \qquad \text {for }\eta \in B_{\bar{t}}(0). \end{aligned}$$

Instead of proving (4.20), we prove a slightly different equivalent statement: for every $\alpha >0$ there exist $\bar{t}\in (0,1)$ and $\bar{k} \in \mathbb {N}$ such that

4.21 $$\begin{aligned} \int _{F \times R} |f_{k}(x+ \eta ,\xi )-f_{k}(x,\xi )|\, \text {d}x \, \text {d}\xi \le \alpha \qquad \text {for }\eta \in B_{\bar{t}}(0)\text { and }k \ge \overline{k}. \end{aligned}$$

For every $\eta \in B_1(0)$, $\xi \in \mathbb {R}^n$ and $j =1,2,\dotsc$, we define $\xi _\eta ^j \in \mathbb {R}^n$ as

$$\begin{aligned} \xi _\eta ^j:= \xi + \frac{1}{j} \eta . \end{aligned}$$

We remark that by Lemma 4.1 for every $\eta \in B_1(0)$ and $j \in \mathbb {N}$ we have that

4.22 $$\begin{aligned}&\sup _{e \in \mathbb {R}^n}\int _{R} \frac{|e|}{1+|e\cdot \xi _\eta ^j|^2} \, \text {d}\xi \le \sup _{e \in \mathbb {R}^n }\int _{B_4(0)\setminus B_{1/4}(0)} \frac{|e|}{1+|e \cdot \xi |^2} \, \text {d}\xi \le C \end{aligned}$$

for some positive constant *C* only depending on the dimension *n*.

Let us now fix $\overline{j} \ge 4$ such that $\overline{j} \ge 4|F|C/\alpha$. We repeatedly apply the triangle inequality to obtain for $t \in [0, 1]$

4.23 $$\begin{aligned}&\left| f_{k}(x+ t \eta , \xi ) - f_{k}( x , \xi ) \right| \nonumber \\&\quad \le |f_{k}( x + t \eta , \xi )- f_{k}( x + t \eta , \xi _\eta ^{\overline{j}} )|+ |f_{k}( x + t \eta , \xi _\eta ^{\overline{j}} ) - f_{k} (x - \overline{j} t \xi , \xi _\eta ^{\overline{j}} )| \nonumber \\&\qquad +|f_{k}( x - \overline{j} t\xi , \xi _\eta ^{\overline{j}} ) - f_{k}( x - \overline{j} t \xi , \xi ) | + | f_{k} ( x - \overline{j}t \xi , \xi ) - f_{k} ( x , \xi ) |. \end{aligned}$$

The first term in (4.23) is bounded as follows

4.24 $$\begin{aligned}&|f_{k}( x + t \eta , \xi )- f_{k}( x + t \eta , \xi _\eta ^{\overline{j}} )| \nonumber \\&\qquad = |\tau (u_k(x + t \eta ) \cdot \xi ) - \tau ( u_k( x + t \eta ) \cdot \xi _\eta ^{\overline{j}} ) | = \left| \int _{u_k ( x + t \eta )\cdot \xi }^{u_k( x + t \eta )\cdot \xi _\eta ^{\overline{j}} } \frac{\text {d}s}{1+s^2} \right| \nonumber \\&\qquad \le \max \Big \{ \frac{1}{1+|u_k(x+t\eta )\cdot \xi |^2},\frac{1}{1+|u_k(x+t\eta )\cdot \xi _\eta ^{\overline{j}} |^2} \Big \} \Big |u_k(x+t\eta )\cdot ( \xi _\eta ^{\overline{j}} - \xi ) \Big |\nonumber \\&\qquad \le \frac{1}{\overline{j}} \max \Big \{ \frac{1}{1+|u_k(x+t\eta )\cdot \xi |^2},\frac{1}{1+|u_k(x+t\eta )\cdot \xi _\eta ^{\overline{j}}|^2} \Big \} |u_k(x+t\eta )|. \end{aligned}$$

Thanks to (4.22), we deduce from (4.24) that

4.25 $$\begin{aligned} \begin{aligned} \int _{R} |f_{k}( x + t \eta , \xi )- f_{k}( x + t \eta , \xi _\eta ^{\overline{j}} )| \, \text {d}\xi \le \frac{1}{\overline{j}}C \, . \end{aligned} \end{aligned}$$

for the same constant *C* defined in (4.22). Hence, we infer from (4.25) and from the choice of $\overline{j}$ that for every $x \in F$, $t \in [0, 1]$, $k \in \mathbb {N}$, and $\eta \in B_1(0)$, it holds

4.26 $$\begin{aligned} \int _{R} | f_{k}(x + t\eta , \xi ) - f_{k} (x + t\eta , \xi ^{\overline{j}}_{\eta }) | \, \text {d}\xi \le \frac{\alpha }{4|F|}. \end{aligned}$$

The very same argument allows us to bound the third term on the right-hand side of (4.23) for every $x \in F$, $t \in [0, 1]$, $k \in \mathbb {N}$, and $\eta \in B_1(0)$ as

4.27 $$\begin{aligned} \int _{R} | f_{k} ( x -\overline{j} t \xi , \xi _\eta ^{\overline{j}} ) - f_{k} ( x - \overline{j} t \xi , \xi )| \, \text {d}\xi \le \frac{\alpha }{4|F|}. \end{aligned}$$

For the second term on the right-hand side of (4.23) we have

$$\begin{aligned}&\int _{R} \int _{F}| f_{k} ( x + t \eta , \xi _\eta ^{\overline{j}} ) - f_{k} ( x - \overline{j} t \xi , \xi _\eta ^{\overline{j}} ) | \text {d}x \, \text {d}\xi \\&= \int _{R} \int _{F - \overline{j} t\xi }|f_{k} ( x + t \overline{j} \xi _\eta ^{\overline{j}} , \xi _\eta ^{\overline{j}} ) -f_{k} ( x , \xi _\eta ^{\overline{j} } ) | \text {d}x \,\text {d}\xi \\&\le \int _{E \times R} |\tau (u_{k} ( x + t \overline{j} \xi _\eta ^{\overline{j}} )\cdot \xi _\eta ^{\overline{j}} ) -\tau (u_{k} (x) \cdot \xi _\eta ^{\overline{j}} ) | \text {d}x \, \text {d}\xi , \end{aligned}$$

if $\overline{j} t \ll 1$, since $F \Subset E$. We apply the change of variable $\xi = \xi ^{\overline{j}}_{\eta }$ to the last integral above to obtain

$$\begin{aligned} \int _{R} \int _{F}&| f_{k} ( x + t \eta , \xi _\eta ^{\overline{j}} ) - f_{k} ( x - \overline{j} t \xi , \xi _\eta ^{\overline{j}} ) | \text {d}x \, \text {d}\xi \\&\le \int _{E \times (B_{4}(0)\setminus B_{1/4}(0))} |\tau (u_{k}( x + t \overline{j} \xi ) \cdot \xi ) - \tau ( u_{k} (x) \cdot \xi ) | \text {d}x \, \text {d}\xi \,. \end{aligned}$$

Recall that, by Lemma 4.4, for $t \in [0,1)$ and $\xi \in B_{4}(0)\setminus B_{1/4}(0)$ such that $E + t \overline{j} \xi \subset \Omega$ we have

4.28 $$\begin{aligned} \int _{E} |\tau (u_{k}(x + t \overline{j} \xi ) \cdot \xi ) - \tau ( u_{k}(x) \cdot \xi ) | \text {d}x \le C_{E} t \overline{j} (1 + F_{ t \overline{j} ,\xi }(u_k, E) ) . \end{aligned}$$

Choose $t_{\alpha } \in (0, 1)$ sufficiently small so that

4.29 $$\begin{aligned}&F \subset E + \overline{j} t \xi \subset \Omega \qquad \text {for every }\xi \in (B_{4}(0)\setminus B_{1/4}(0))\text { and every }t \in [0, t_{\alpha }],\nonumber \\&C_E \bar{j} t_{\alpha } (1+M)\le \frac{\alpha }{4}. \end{aligned}$$

Let $\bar{k}\in \mathbb {N}$ sufficiently large and let us set $t_k:= i_k \varepsilon _k \in (\frac{t_{\alpha }}{2}, t_{\alpha })$ for $k \ge \bar{k}$ and for some $i_k\in \mathbb {N}$. Thanks to (4.29), we apply (4.28) for $t = t_{k}$ and Lemma 4.5 with the choice $m = \overline{j} i_{k}$, obtaining

$$\begin{aligned} \int _{E \times (B_{4}(0)\setminus B_{1/4}(0)) }&|\tau (u_{k}(x + \overline{j} t_k \xi ) \cdot \xi ) - \tau ( u_{k} (x) \cdot \xi ) | \text {d}x \,\text {d}\xi \\&=\int _{E \times (B_{4}(0)\setminus B_{1/4}(0)) } | \tau ( u_{k} ( x + \overline{j} i_k \varepsilon _k\xi )\cdot \xi ) - \tau ( u_{k}(x) \cdot \xi )| \text {d}x \,\text {d}\xi \\&\le C_{E} \overline{j} i_k\varepsilon _k \int _{ (B_{4}(0)\setminus B_{1/4}(0)) } ( 1 + F_{ \overline{j} i_k \varepsilon _k,\xi }(u_k, E )) \text {d}\xi \\&\phantom {\int _{E}}\le C_E \overline{j} i_k \varepsilon _k(1+\mathcal {F}^1_{\varepsilon _k}(u_k, \Omega )). \end{aligned}$$

Summarizing we have thus obtained for every $k \ge \bar{k}$ and every $\eta \in B_{1}(0)$

4.30 $$\begin{aligned} \int _{F \times R} |f_{k}(x + t_k \eta , \xi _\eta ^{\overline{j}} ) - f_{k}(x- \overline{j} t_k\xi , \xi _\eta ^{\overline{j}} ) | \text {d}x \, \text {d}\xi&\le C_E \overline{j} i_k \varepsilon _k(1+\mathcal {F}^1_{\varepsilon _k}(u_k, \Omega )) \nonumber \\&\le C_E\overline{j} t_{\alpha } ( 1 + M) \le \frac{\alpha }{4}. \end{aligned}$$

From the very same argument, we infer that for every $k \ge \bar{k}$ and every $\eta \in B_{1}(0)$

4.31 $$\begin{aligned} \int _{F \times R} |f_{k}(x - \overline{j} t_k\xi ,\xi ) - f_{k} ( x , \xi ) | \text {d}x \, \text {d}\xi \le C_E \overline{j} i_k\varepsilon _k(1+\mathcal {F}^1_{\varepsilon _k}(u_k, \Omega )) \le \frac{\alpha }{4} \,. \end{aligned}$$

Combining (4.26), (4.27), (4.30), (4.31), we have shown that for every $k \ge \bar{k}$ and every $\eta \in B_{1}(0)$ it holds

4.32 $$\begin{aligned} \int _{F \times R } f_{k} (x + t_{k} \eta , \xi ) - f(x , \xi ) \, \textrm{d}x \, \textrm{d}\xi \le \alpha \,, \end{aligned}$$

where we recall that $t_{k} \in (\frac{t_{\alpha }}{2}, t_{\alpha })$. Finally, setting $\bar{t}:= t_{\alpha }/2$, (4.32) yields

$$\begin{aligned} \int _{R} \int _{F}|f_{k}(x+\eta ,\xi )-f_{k}(x,\xi )| \text {d}x \, \text {d}\xi \le \alpha , \end{aligned}$$

for every $k \ge \overline{k}$ and every $\eta \in B_{\overline{t}} (0)$, which is precisely (4.21).

**Step 2:** Now we prove that for every $\alpha >0$ there exists $\bar{t} \in (0, 1)$ such that

4.33 $$\begin{aligned} \int _{F \times R} |f_{k}(x,\xi + \eta )-f_{k}(x,\xi )| \,\text {d}x \,\text {d}\xi \le \alpha \qquad \text {for }\eta \in B_{\bar{t}}(0)\text { and }k \in \mathbb {N}. \end{aligned}$$

Fix $\eta \in \mathbb {S}^{n-1}$ and let $t \in (0,1).$ By arguing as in (4.24) we have that for $x \in F$

$$\begin{aligned}&|\tau (u_k(x) \cdot (\xi +t\eta )) - \tau (u_k(x) \cdot \xi )| \\&\le t \, \max \Big \{ \frac{1}{1+|u_k(x)\cdot \xi |^2},\frac{1}{1+|u_k(x)\cdot (\xi + t \eta )|^2} \Big \} |u_k(x) \cdot \eta |. \end{aligned}$$

From this last inequality we deduce from Lemma 4.1 that

4.34 $$\begin{aligned} \int _{R}|\tau (u_k(x) \cdot (\xi +t\eta )) - \tau (u_k(x) \cdot \xi )|\,\text {d}\xi \le C t\,, \end{aligned}$$

for a positive constant *C* only depending on the dimension *n*. By (4.34) there exists $\bar{t} \in (0, 1)$ such that for every $x \in F$, $k \in \mathbb {N}$, $\eta \in \mathbb {S}^{n-1}$, and every $t \in [0, \bar{t}]$

4.35 $$\begin{aligned} \int _{R} |f_{k} (x, \xi + t\eta ) - f_{k} (x, \xi ) | \, \text {d}\xi \le \alpha . \end{aligned}$$

Eventually, (4.35) leads to (4.33).

Thanks to (4.20) and (4.33), it is not difficult to see that we are in position to apply Fréchet-Kolmogorov Theorem (see, e.g., [11, Theorem 4.26]) and obtain that the sequence $\{f_k\}_{k \in \mathbb {N}}$ is relatively compact in $L^1(F \times R)$ for every open set *F* compactly contained in $\Omega$. Therefore, up to passing to a subsequence, we have $f_k \rightarrow f_{\infty }$ as $k \rightarrow \infty$ strongly in $L_{loc}^1(\Omega \times R)$. By a diagonal argument we can possibly pass to another subsequence such that

4.36 $$\begin{aligned} f_k \rightarrow f_{\infty } \qquad \text {pointwise a.e. in }\Omega \times R. \end{aligned}$$

From (4.36), we deduce the existence of an orthonormal basis $\{\eta _1,\ldots ,\eta _n\}$ of $\mathbb {R}^n$ such that

$$\begin{aligned} \tau (u_k \cdot \eta _i) \rightarrow f^i_\infty \qquad \text {pointwise a.e. in }\Omega \text { for every }i=1,\dotsc ,n\text { as }k \rightarrow \infty , \end{aligned}$$

for some measurable function $$f^i_\infty :\Omega \rightarrow [-\pi /2,\pi /2]$$. Since $$\arctan $$ is a diffeomorphism with its image, we deduce the existence of a measurable function $$u:\Omega \rightarrow \mathbb {R}^n$$ such that $$ u=0$$ on *A* and

4.37 $$\begin{aligned} u_k \rightarrow u \qquad&\text {a.e. in }\Omega \setminus A\text { as }k \rightarrow \infty \end{aligned}$$

4.38 $$\begin{aligned} |u_k| \rightarrow \infty \qquad&\text {a.e. in }A\text { as }k \rightarrow \infty , \end{aligned}$$

where *A* is the set defined in (1.6). Eventually, up to passing to a subsequence, we also assume that $u_k \rightarrow u$ in measure in $\Omega \setminus A$. We further remark that

4.39 $$\begin{aligned} x \in A \iff \lim _{k \rightarrow \infty } |u_k(x)\cdot \xi |=\infty \quad \text {for a.e. } \xi \in \mathbb {R}^n. \end{aligned}$$

**Step 3:** Let $\lambda >0$ and let $\tau _\lambda :\mathbb {R}\rightarrow [-\lambda ,\lambda ]$ be a strictly increasing function such that $\tau '_\lambda \le 1$, $\lim _{t \rightarrow \pm \infty } \tau _\lambda (t)=\pm \lambda$, and $\tau _{\lambda }(t)=t$ for $t \in [-\lambda /2,\lambda /2]$. Fix $\xi \in \mathbb {R}^n$, and let $y \in \Pi ^{\xi }$. For every $\lambda >0$, we want to construct suitable modifications $\{v_{j,\lambda }\}_{j \in \mathbb {N}} \subset \text {SBV}(\Omega _y^\xi )$ of $(\hat{u}_{k})_{y,\lambda }^{\xi }(t):=\tau _\lambda (u_k(y+t \xi )\cdot \xi )$, as in [31, Theorem 3.4, Step 2], such that

4.40 $$\begin{aligned} v_{j,\lambda } \rightarrow \hat{u}_{y,\lambda }^{\xi } \text { in } L^1_{loc}(\Omega _y^\xi )\text { as } j \rightarrow \infty , \end{aligned}$$

and

4.41 $$\begin{aligned} \liminf _{k \rightarrow \infty } F_{\varepsilon _k}\big ((\hat{u}_{k})_{y,\lambda }^{\xi },I \cap (I-\varepsilon _k)\big ) \ge \frac{\pi }{2}\mathcal{M}\mathcal{S}_{\frac{2}{\pi }}(v_{j,\lambda },I) \end{aligned}$$

for every *j* sufficiently large and every interval $I \Subset \Omega _y^\xi$, where $\hat{u}_{y,\lambda }^{\xi }(t):=\tau _\lambda (u(y+t\xi )\cdot \xi )$ for $y+t\xi \in \Omega \setminus A$ and $\hat{u}_{y,\lambda }^{\xi }(t) = \pm \lambda$ for $y+t \xi \in A$.

We notice that by applying Fubini’s Theorem for a.e. $\xi$ we have

$$\begin{aligned} \mathcal {L}^n(\{x \in A: \liminf _{k \rightarrow \infty } |u_k(x)\cdot \xi |<\infty \})=0. \end{aligned}$$

Then thanks to (4.37)–(4.38), up to a subsequence which does not depend on $\xi$ and *y* we have (for every $\lambda >0$)

4.42 $$\begin{aligned}&(\hat{u}_{k})_{y,\lambda }^{\xi } \rightarrow \hat{u}_{y,\lambda }^{\xi } \qquad \text {in }L^1_{loc}((\Omega \setminus A)_y^\xi )\text { for every }\xi \text { and for }\mathcal {H}^n\text {-a.e. }y \in \Pi ^\xi ,\end{aligned}$$

4.43 $$\begin{aligned}&|(\hat{u}_{k})_{y,\lambda }^{\xi }| \rightarrow |\hat{u}_{y,\lambda }^{\xi }| =\lambda \qquad \text {in }L^1_{loc}(A_y^\xi )\text { for a.e. }\xi , \text { for }\mathcal {H}^{n-1}\text {-a.e.}~y \in \Pi ^\xi , \end{aligned}$$

as $k \rightarrow \infty$. We further observe that, since $0 \le \tau '_\lambda \le 1$, then $|\tau _\lambda (t)- \tau _\lambda (t')| \le |t -t'|$ for every $t,t' \in \mathbb {R}$, leading to

4.44 $$\begin{aligned} F_\varepsilon (f,B) \ge F_\varepsilon (\tau _\lambda (f),B), \end{aligned}$$

for every $\varepsilon >0$, every measurable set $B \subset \mathbb {R}$, and every measurable function $f :B \rightarrow \mathbb {R}$.

We extend the function $\hat{u}^\xi _{y,\lambda }$ to $\mathbb {R}$ in such a way that $\hat{u}^\xi _{y,\lambda }(t)=0$ for $t \in \mathbb {R}\setminus \Omega _y^\xi$. Let $a \in \mathbb {R}$ satisfy conditions (*i*) and (*ii*) of Lemma 4.3 for $\hat{u}^\xi _{y,\lambda }$, and let $I_j^z:=[a+\frac{z}{j},a+\frac{z+1}{j}]\Subset \mathbb {R}.$ We define $v_{j,\lambda }$ in every interval $I_j^z$ in the following way:

- If $j(\hat{u}_{y,\lambda }^{\xi } (a+\frac{z+1}{j})-\hat{u}_{y,\lambda }^{\xi }(a+\frac{z}{j}))^2\le \frac{\pi }{2},$ then $v_{j,\lambda }$ is the affine function that coincides with $\hat{u}_{y,\lambda }^{\xi }$ at the endpoints of $I_j^z;$
- if $j(\hat{u}_{y,\lambda }^{\xi }(a+\frac{z+1}{j})-\hat{u}_{y,\lambda }^{\xi }(a+\frac{z}{j}))^2> \frac{\pi }{2},$ then $v_{j,\lambda }$ is the piecewise constant function that coincides with $\hat{u}_{y,\lambda }^{\xi }$ at the endpoints of $I_j^z$ and has a unique discontinuity in the middle point of the interval.

Then by Lemma 4.3, up to subsequences, (4.40) holds. Moreover, $v_{j,\lambda } \in \text {SBV}(\Omega _y^\xi )$ and

$$\begin{aligned} \frac{\pi }{2} \mathcal{M}\mathcal{S}_{\frac{2}{\pi }}(v_{j;\lambda },I_j^z)=\min \left\{ \frac{\pi }{2},j \left( \hat{u}_{y,\lambda }^{\xi }\left( a+\frac{z+1}{j}\right) -\hat{u}_{y,\lambda }^{\xi }\left( a+\frac{z}{j}\right) \right) ^2 \right\} \end{aligned}$$

for every $j \in \mathbb {N}$ and $z \in \mathbb {Z}$ such that $I_j^z \Subset \Omega _y^\xi$.

Let $I \subset \Omega _y^\xi$ be an interval and $F \Subset I \cap (I-\varepsilon _k)$ for *k* sufficiently large, note that such *F* exists since $I \cap (I -\varepsilon _k) \rightarrow I$ as $k \rightarrow \infty$. We notice that for every *j* sufficiently large, depending on *F*, every intervals $I_j^z$ such that $I_j^z \subset I \cap (I-\varepsilon _k)$ also satisfy $F \subset \cup _{z} I_j^z$. By virtue of (4.42) and (4.43) we can make use of Lemma 4.2 in $I_j^z$ and infer, for a.e. $\xi \in \mathbb {R}^n$ and for $\mathcal {H}^{n-1}$-a.e. $y \in \Pi ^\xi$, that

$$\begin{aligned} \liminf _{k \rightarrow \infty } F_{\varepsilon _k}((\hat{u}_{k})_{y,\lambda }^{\xi },I_j^z) \ge \frac{\pi }{2} \mathcal{M}\mathcal{S}_{\frac{2}{\pi }}(v_{j,\lambda },I_j^z). \end{aligned}$$

To prove (4.41) we sum over all *z* such that $I_j^z \subset I \cap (I-\varepsilon _k)$ for every *j* sufficiently large

$$\begin{aligned} \liminf _{k \rightarrow \infty } F_{\varepsilon _k}((\hat{u}_{k})_{y,\lambda }^{\xi },I \cap (I-\varepsilon _k)) \ge \frac{\pi }{2}\mathcal{M}\mathcal{S}_{\frac{2}{\pi }}(v_{j,\lambda },F). \end{aligned}$$

From the semicontinuity of the Mumford-Shah functional with respect to the $L_{loc}^1$-convergence, taking the limit as $j \rightarrow \infty$ and then as $F \nearrow I$, we obtain

4.45 $$\begin{aligned} \liminf _{k \rightarrow \infty } F_{\varepsilon _k}\big ((\hat{u}_{k})_{y,\lambda }^{\xi },I \cap (I- \varepsilon _k)\big ) \ge \frac{\pi }{2} \mathcal{M}\mathcal{S}_{\frac{2}{\pi }}(\hat{u}_{y,\lambda }^{\xi },I). \end{aligned}$$

We recall that

$$\begin{aligned} \mathcal {F}^1_{\varepsilon }(u,\Omega )&= \sup _{\mathscr {B}}\sum _{B \in \mathscr {B}}\int _{\frac{\Omega -\Omega }{\varepsilon }} F_{\varepsilon ,\xi }(u,B) e^{-|\xi |^2} \text {d}\xi \\&=\sup _{\mathscr {B}}\sum _{B \in \mathscr {B}}\int _{\frac{\Omega -\Omega }{\varepsilon }} \left( \int _{\Pi ^\xi }F_{\varepsilon }(\hat{u}_y^\xi ,B_y^\xi \cap (B-\varepsilon \xi )_y^\xi ) \text {d}\mathcal {H}^{n-1}(y) \right) |\xi | e^{-|\xi |^2} \text {d}\xi . \end{aligned}$$

From (4.44), Fatou’s Lemma, and (1.5), we get

$$\begin{aligned} \begin{aligned} \sup _{\mathscr {B}}&\sum _{B \in \mathscr {B}}\int _{\mathbb {R}^n} \int _{\Pi ^\xi } \liminf _{k \rightarrow \infty } F_{\varepsilon _k}((\hat{u}_k)_{y,\lambda }^\xi ,B_y^\xi \cap (B-\varepsilon _k\xi )_y^\xi ) |\xi | e^{-|\xi |^2}\text {d}\mathcal {H}^{n-1}(y)\, \text {d}\xi \\&\le \sup _{\mathscr {B}}\sum _{B \in \mathscr {B}}\int _{\mathbb {R}^n} \int _{\Pi ^\xi } \liminf _{k \rightarrow \infty } F_{\varepsilon _k}((\hat{u}_k)_{y}^\xi ,B_y^\xi \cap (B-\varepsilon _k\xi )_y^\xi )|\xi | e^{-|\xi |^2} \text {d}\mathcal {H}^{n-1}(y) \,\text {d}\xi \\&\le \liminf _{k \rightarrow \infty } \sup _{\mathscr {B}}\sum _{B \in \mathscr {B}} \int _{\frac{\Omega -\Omega }{\varepsilon _k}} \int _{\Pi ^\xi } F_{\varepsilon _k}((\hat{u}_k)_{y}^\xi ,B_y^\xi \cap (B-\varepsilon _k\xi )_y^\xi ) |\xi | e^{-|\xi |^2}\text {d}\mathcal {H}^{n-1}(y)\, \text {d}\xi \\&\le \liminf _{k \rightarrow \infty }\mathcal {F}^1_{\varepsilon _k}(u_k,\Omega ) \le M, \end{aligned} \end{aligned}$$

which yields for every open ball $B \subset \Omega$

4.46 $$\begin{aligned} \!\!\! \liminf _{k \rightarrow \infty } F_{\varepsilon _k}((\hat{u}_k)_{y,\lambda }^\xi ,B_y^\xi \cap (B-\varepsilon _k\xi )_y^\xi ) \le C \quad \text {for a.e. } \xi \in \mathbb {R}^n, \text { for }\mathcal {H}^{n-1}\text {-a.e.}~y \in \Pi ^\xi \end{aligned}$$

where the constant $C>0$ may depend on $\xi$ and *y* but not on $\lambda$ and *B*. The above inequality combined with (4.45) implies that $\hat{u}_{y,\lambda }^\xi \in \text {SBV}_{loc}(\Omega _y^\xi )$ for a.e. $\xi \in \mathbb {R}^n$ and $\mathcal {H}^{n-1}$-a.e. $y \in \Pi ^\xi$.

**Step 4:** We claim that for a.e. $\xi \in \mathbb {R}^n$, for $\mathcal {H}^{n-1}$-a.e. $y \in \Pi ^\xi$, and for every bounded interval $I \subset \Omega _y^\xi$ we have

4.47 $$\begin{aligned} \mathcal {H}^0(A_y^\xi \cap I) \ne 0 \implies \mathcal {H}^0(J_{\hat{u}_{y,\lambda }^{\xi }}\cap I) \ne 0 \text { or } I \subset A_y^\xi . \end{aligned}$$

If $I\subset A_y^\xi$ we have nothing to prove. Let us assume that there exists $s \in I \setminus A_y^\xi .$ By contradiction, assume $\mathcal {H}^0(J_{\hat{u}_{y,\lambda }^{\xi }}\cap I)=0.$ For a.e. $\xi \in \mathbb {R}^n$, for $\mathcal {H}^{n-1}$-a.e. $y \in \Pi ^\xi$, by (4.45) and (4.46) we get

4.48 $$\begin{aligned} \int _{I} |\nabla \hat{u}_{y,\lambda }^{\xi }(t)|^2 \, \text {d}t \le C . \end{aligned}$$

Since $\hat{u}_{y,\lambda }^{\xi }$ belongs to $\text {SBV}(I)$ and since $\mathcal {H}^0(J_{\hat{u}_{y,\lambda }^{\xi }}\cap I)=0$, it is absolutely continuous in *I*. Hence, for $\bar{t} \in I$ we have

$$\begin{aligned} |\hat{u}_{y,\lambda }^{\xi }(\bar{t})|&=\left| \hat{u}_{y,\lambda }^{\xi }(s)+\int ^t_s \nabla \hat{u}_{y,\lambda }^{\xi }(t) \, \text {d}t \right| \\&\le |\hat{u}_{y,\lambda }^{\xi }(s)|+ \bigg | \int ^t_s |\nabla \hat{u}_{y,\lambda }^{\xi }(t)| \,\text {d}t \bigg | \\&\le |\hat{u}_{y,\lambda }^{\xi }(s)|+\text {lenght}(I)^{1/2} \left| \int ^t_s |\nabla \hat{u}_{y,\lambda }^{\xi }(t)|^2 \,\text {d}t \right| ^{1/2} \le C, \end{aligned}$$

for every $\bar{t} \in I$, where the last inequality follows from (4.48) and the fact that $s \in I \setminus A_y^\xi$. Since $\mathcal {H}^0(A_y^\xi \cap I) \ne 0$ and (4.39) holds, there exists $\bar{t} \in A_y^\xi \cap I$ such that $|\hat{u}_{y,\lambda }^{\xi }(\bar{t})|>C$ for every $\lambda >0$ sufficiently large, which is a contradiction. This concludes the proof of (4.47).

**Step 5:** Let $I \subset \Omega _y^\xi$ be a bounded interval. We claim that, for a.e. $\xi \in \mathbb {R}^n$ and for $\mathcal {H}^{n-1}$-a.e. $y \in \Pi ^\xi$, the set $A_y^\xi$ is a finite union of intervals and

4.49 $$\begin{aligned} \frac{\pi }{2}\mathcal {H}^0(\partial ^* A_y^\xi \cap I)\le \liminf _{k \rightarrow \infty } F_{\varepsilon _k}((\hat{u}_k)_{y,\lambda }^\xi ,I\cap (I-\varepsilon _k)). \end{aligned}$$

Moreover, *A* is a set of finite perimeter.

Denote by $(A_y^\xi )^d$ the points of density *d* of $A_y^\xi .$ We want to prove that $(A_y^\xi )^1$ is an open set. By contradiction, suppose that there exist $t \in (A_y^\xi )^1$ and a sequence $\{s_j\}_{j \in \mathbb {N}} \subset \Omega _y^\xi \setminus (A_y^\xi )^1$ such that $s_j \rightarrow t.$ Since almost every points of $\Omega _y^\xi \setminus (A_y^\xi )^1$ belong to $(A_y^\xi )^0$, we find another sequence $\{t_j\}_{j \in \mathbb {N}} \subset (A_y^\xi )^0$ such that $t_j \rightarrow t$. Without loss of generality we assume $t^0_j< t^0_{j+1}$ for every $j \in \mathbb {N}.$ By definition of points of density zero, for every *j*, there exists a point $t^1_j \in [t^0_j,t^0_{j+1})$ of density one. By the previous step, there exists another increasing sequence $t_j \in [t^0_j,t^0_{j+1}) \cap J_{\hat{u}_{y,\lambda }^{\xi }}$. Since the sequence $\{t_j\}_{j \in \mathbb {N}}$ is strictly increasing, there exist disjoint open intervals $I_j$ such that $t_j \in I_j$. Now, by applying (4.45) and (4.46) on every $I_j$ we obtain $\sum _j 1\le \sum _j \mathcal {H}^0(J_{\hat{u}_{y,\lambda }^{\xi }} \cap I_j)\le C,$ which is a contradiction. Hence, $(A^{\xi }_{y})^{1}$ is open.

From now on we assume that $A_y^\xi$ coincides with $(A_y^\xi )^1$. Suppose that $|I \setminus A_y^{\xi }|>0$, otherwise there is nothing to prove. Let *K* be a connected component of $A_y^\xi \cap I$, and let $t_1 \in K$, $t_2 \in I \setminus A_y^\xi .$ Then, by Step 4, there exists $t \in J_{\hat{u}_{y,\lambda }^{\xi }} \cap I$ between $t_1$ and $t_2$. Using a similar argument as above, we prove that $\mathcal {H}^0(J_{\hat{u}_{y,\lambda }^{\xi }} \cap I)$ is greater than or equal to the number of connected components of $A_y^\xi \cap I$ and is uniformly bounded, from which it follows that $A_y^\xi$ is the union of a finite number of intervals. Finally, (4.49) is a consequence of $\mathcal {H}^0(\partial ^*A_y^\xi \cap I)\le \mathcal {H}^0(J_{\hat{u}_{y,\lambda }^\xi } \cap I)$ and of (4.45).

To conclude we need to prove that *A* is of finite perimeter. Multiplying (4.49) by $|\xi | e^{-|\xi |^2}$ and then integrating in $\xi \in \mathbb {R}^n$ and $y \in \Pi ^\xi$ we obtain for every ball $B \subset \Omega$

$$\begin{aligned} C(n) \mathcal {H}^{n-1}(\partial ^* A \cap B)&\le \int _{\mathbb {R}^n} \Big ( \int _{\Pi ^\xi } \mathcal {H}^0(\partial ^* A_y^\xi \cap B_y^\xi ) \text {d}\mathcal {H}^{n-1}(y) \Big ) |\xi | e^{-|\xi |^2}\text {d}\xi \\&\le \int _{\mathbb {R}^n} \Big (\int _{\Pi ^\xi } \liminf _{k \rightarrow \infty } F_{\varepsilon _k}((\hat{u}_k)_{y,\lambda }^\xi ,B_y^\xi \cap (B_y^\xi -\varepsilon _k)) \text {d}\mathcal {H}^{n-1}(y) \Big ) |\xi | e^{-|\xi |^2} \text {d}\xi \\&\le \liminf _{k \rightarrow \infty } \mathcal {F}^1_{\varepsilon _k}(u_k,B) , \end{aligned}$$

where the last inequality follows from Fatou’s Lemma. This proves in particular that *A* has locally finite perimeter in $\Omega$. By Besicovitch covering Theorem there exist a dimensional constant *j*(*n*) and $\mathcal {B}_1, \ldots , \mathcal {B}_{j(n)}$ countable families of pairwise disjoint open balls contained in $\Omega$, such that $\Omega \subset \cup _{i=1}^{j(n)} \cup _{B \in \mathcal {B}_i} B$. Hence

$$\begin{aligned} C(n)\mathcal {H}^{n-1}(\partial ^* A)&\le \sum _{i=1}^{j(n)} \sum _{B \in \mathcal {B}_i} C(n) \mathcal {H}^{n-1}(\partial ^* A \cap B) \\&\le \sum _{i=1}^{j(n)} \sum _{B \in \mathcal {B}_i}\liminf _{k \rightarrow \infty } \mathcal {F}^1_{\varepsilon _k}(u_k,B) \\&\le j(n) \liminf _{k \rightarrow \infty } \sum _{B \in \mathcal {B}_i}\mathcal {F}^1_{\varepsilon _k}(u_k,B) \\&\le j(n) \liminf _{k \rightarrow \infty }\mathcal {F}^1_{\varepsilon _k}(u_k,\Omega ) <\infty , \end{aligned}$$

where, in the last but one inequality above, we used Remark 2.12.

**Step 6:** We conclude by proving that $u \in \text {GSBD} (\Omega )$ and that (1.7) holds.

Fix $\mathscr {B}$ be any finite family of disjoint balls in $\Omega$ and let $B \in \mathscr {B}$. For a.e. $\xi \in \mathbb {R}^n$, for $\mathcal {H}^{n-1}$-a.e. $y \in \Pi ^\xi$, for every interval $I \subset B_y^\xi$ of the form $I=(t-\delta ,t+\delta )$ for $t \in \partial ^* A_y^\xi$ and $\delta >0$, by (4.44), (4.45) and (4.49), we have

$$\begin{aligned} \liminf _{k \rightarrow \infty }&\ F_{\varepsilon _k}((\hat{u}_k)_y^\xi ,B_y^\xi \cap (B-\varepsilon _k\xi )_y^\xi ) \\&\ge \liminf _{k \rightarrow \infty } F_{\varepsilon _k}((\hat{u}_k)_y^\xi ,I \cap (B-\varepsilon _k\xi )_y^\xi ) + \liminf _{k \rightarrow \infty } F_{\varepsilon _k}((\hat{u}_k)_y^\xi ,B_y^\xi \setminus I \cap (B-\varepsilon _k\xi )_y^\xi ) \\&\ge \liminf _{k \rightarrow \infty } F_{\varepsilon _k}((\hat{u}_k)_{y,\lambda }^\xi ,I \cap (B-\varepsilon _k\xi )_y^\xi ) + \liminf _{k \rightarrow \infty } F_{\varepsilon _k}((\hat{u}_k)_{y,\lambda }^\xi ,B_y^\xi \setminus I \cap (B-\varepsilon _k\xi )_y^\xi ) \\&\ge \frac{\pi }{2}\mathcal {H}^0(\partial ^*A_y^\xi \cap I)+\frac{\pi }{2} \mathcal{M}\mathcal{S}_{\frac{2}{\pi }}(\hat{u}^{\xi }_{y,\lambda },B_y^\xi \setminus I) \\&=\frac{\pi }{2}\mathcal {H}^0(\partial ^*A_y^\xi \cap I) + \frac{\pi }{2}\mathcal {H}^0(J_{\hat{u}^\xi _{y,\lambda }}\setminus I) + \int _{B_y^\xi \setminus I}|\nabla \hat{u}^{\xi }_{y,\lambda }(t)|^2 \,\text {d}t. \end{aligned}$$

Since the above inequality holds for every $\delta >0$ and $t \in \partial ^* A_y^\xi$, we get

$$\begin{aligned} \liminf _{k \rightarrow \infty } F_{\varepsilon _k}((\hat{u}_k)_y^\xi ,B_y^\xi \cap (B-\varepsilon _k\xi )_y^\xi )\ge \frac{\pi }{2} \mathcal {H}^0(\partial ^*A_y^\xi \cup J_{\hat{u}_{y,\lambda }^\xi })+\int _{B_y^\xi }|\nabla \hat{u}^{\xi }_{y,\lambda }(t)|^2 \,\text {d}t. \end{aligned}$$

Since $\tau _\lambda (t)=t$ for $t \in [-\lambda /2,\lambda /2]$ and $J_{\hat{u}^\xi _{y,\lambda }} =J_{\hat{u}^\xi _{y}}$, we infer that

$$\begin{aligned} \liminf _{k \rightarrow \infty }&F_{\varepsilon _k}((\hat{u}_k)_y^\xi ,B_y^\xi \cap (B-\varepsilon _k\xi )_y^\xi ) \ge \frac{\pi }{2} \mathcal {H}^0(\partial ^*A_y^\xi \cup J_{\hat{u}_{y,\lambda }^\xi })+\int _{B_y^\xi }|\nabla \hat{u}^{\xi }_{y,\lambda }(t)|^2 \,\text {d}t\\&\ge \frac{\pi }{2} \mathcal {H}^0(\partial ^*A_y^\xi \cup J_{\hat{u}_{y}^\xi }) + \int _{\big \{ t \in B_y^\xi :| \hat{u}^{\xi }_{y,\lambda }(t)|\le \lambda /2\big \}}|\nabla \hat{u}^{\xi }_{y}(t)|^2 \,\text {d}t . \end{aligned}$$

Now, by sending $\lambda \rightarrow \infty$ we get for every $c \in \mathbb {R}$

$$\begin{aligned} \begin{aligned} \liminf _{k \rightarrow \infty } F_{\varepsilon _k}((\hat{u}_k)_y^\xi ,B_y^\xi \cap (B-\varepsilon _k\xi )_y^\xi )&\ge \frac{\pi }{2} \mathcal {H}^0(\partial ^*A_y^\xi \cup J_{\hat{u}_{y}^\xi }) +\int _{B_y^\xi }|\nabla \hat{u}^{\xi }_{y}(t)|^2 \,\text {d}t\\&\ge \frac{\pi }{2}\mathcal{M}\mathcal{S}_{\frac{2}{\pi }}(\hat{u}_y^\xi (1-\chi _{A_y^\xi })+c\chi _{A_y^\xi },B_y^\xi ). \end{aligned} \end{aligned}$$

By combining the above inequality with Fatou’s Lemma we deduce

$$\begin{aligned}&\sup _{\mathscr {B}} \sum _{B \in \mathscr {B}}\frac{\pi }{2}\left( \int _{\mathbb {R}^n} \left( \int _{\Pi ^\xi } \mathcal{M}\mathcal{S}_{\frac{2}{\pi }}(\hat{u}_y^\xi (1-\chi _{A_y^\xi })+c \chi _{A_y^\xi },B_y^\xi ) \text {d}\mathcal {H}^{n-1}(y) \right) ^{p}|\xi |^p e^{-|\xi |^2} \text {d}\xi \right) ^{\frac{1}{p}}\\&\le \liminf _{k \rightarrow \infty }\sup _{\mathscr {B}} \sum _{B \in \mathscr {B}}\left( \int _{\frac{\Omega -\Omega }{\varepsilon _k}} \left( \int _{\Pi ^\xi } F_{\varepsilon _k}((\hat{u}_k)_y^\xi ,B_y^\xi \cap (B-\varepsilon _k\xi )_y^\xi )\text {d}\mathcal {H}^{n-1}(y) \right) ^{p}|\xi |^p e^{-|\xi |^2} \text {d}\xi \right) ^{\frac{1}{p}}\\&=\liminf _{k \rightarrow \infty } \mathcal {F}^p_{\varepsilon _k}(u_k,\Omega ) <\infty , \end{aligned}$$

which implies that $\hat{\mu }_{u,p}(\Omega )<\infty$, and hence, by Remark 2.10, also that $u \in \text {GSBV}^{\mathcal {E}}(\Omega ;\mathbb {R}^n)$. Thus by Theorem 3.1 we conclude that $u \in \text {GSBD}(\Omega )$. In addition, recall that, by Lemma 4.6, we have for every $c\in \mathbb {R}$

$$\begin{aligned} \sup _{\mathscr {B}} \sum _{B \in \mathscr {B}}\frac{\pi }{2}&\left( \int _{\mathbb {R}^n} \left( \int _{\Pi ^\xi } \mathcal{M}\mathcal{S}_{\frac{2}{\pi }}(\hat{u}_y^\xi (1-\chi _{A_y^\xi })+c \chi _{A_y^\xi },B_y^\xi ) \text {d}\mathcal {H}^{n-1}(y) \right) ^{p}|\xi |^p e^{-|\xi |^2} \text {d}\xi \right) ^{\frac{1}{p}}\\&=\!\int _{\Omega } \varphi _{p} (e(u_c(1-\chi _A))) \text {d}x + \beta _{p} \mathcal {H}^{n-1}(J_{u_c}), \end{aligned}$$

where $u_c:\Omega \rightarrow \mathbb {R}^n$ is defined as $u_c:=u(1-\chi _A)+c \chi _A$. Since a measure theoretic argument yields that $\partial ^*A \subset J_{u_c}$ for a.e. $c \in \mathbb {R}$, inequality (1.7) follows.

## $\Gamma$-convergence and convergence of quasi-minimisers

This section is devoted to the proof of Theorem 1.2 as well as to the convergence, up to subsequences, of quasi-minimizers of $\mathcal {F}^{p}_{\varepsilon }$ under Dirichlet boundary conditions. The former is performed in Section 5.1, the latter in Section 5.2.

### $\Gamma$-convergence

We start with the following pointwise convergence result following the strategy in [31, Theorem 3.4].

#### Proposition 5.1

Let $I \subset \mathbb {R}$ be an interval and let $u \in \text {GSBV}(I)$. For every $\varepsilon >0$, it holds

5.1 $$\begin{aligned} F_{\varepsilon }(u,I \cap (I-\varepsilon ))\le \frac{\pi }{2}\mathcal{M}\mathcal{S}_{\frac{2}{\pi }}(u,I). \end{aligned}$$

#### Proof

Since *I* is an interval, the set $I \cap (I - \varepsilon )$ is also an interval or it is empty. Assume that it is not empty otherwise there is nothing to prove. Define $A^\varepsilon := \{t \in I \cap (I -\varepsilon ): [t,t+\varepsilon ] \cap J_u \ne \emptyset \}$. Notice that $t \in I \cap (I -\varepsilon )$ implies $[t,t+\varepsilon ] \subset I$. We have

$$ \frac{1}{\varepsilon }\int _{A_\varepsilon } \arctan \bigg (\frac{(u(t+\varepsilon )- u(t))^2}{\varepsilon } \bigg ) \text {d}t \le \frac{\pi }{2 \varepsilon } |A_\varepsilon | \le \frac{\pi }{2} \mathcal {H}^0(J_u)\,. $$

Since $\arctan x \le x$ for $x \ge 0$, we have

$$\begin{aligned}&\frac{1}{\varepsilon }\int _{(I \cap (I-\varepsilon ))\setminus A_\varepsilon } \arctan \bigg (\frac{(u(t+\varepsilon )- u(t))^2}{\varepsilon } \bigg ) \text {d}t \\&\le \frac{1}{\varepsilon } \int _{(I \cap (I-\varepsilon ))\setminus A_\varepsilon } \arctan \bigg (\int _t^{t + \varepsilon } |\nabla u(\tau )|^2\, \text {d}\tau \bigg ) \text {d}t\\&\le \frac{1}{\varepsilon } \int _{(I \cap (I-\varepsilon ))\setminus A_\varepsilon } \int _t^{t + \varepsilon } |\nabla u(\tau )|^2\, \text {d}\tau \,\text {d}t\le \int _{I} |\nabla u(t)|^2 \text {d}t. \end{aligned}$$

By combining the above two inequalities we conclude the proof. $\square$

We are now in a position to conclude the proof of Theorem 1.2.

#### Proof of Theorem 1.2

We notice that the $\Gamma$-liminf inequality is a direct consequence of Theorem 1.1 (cf. (1.7)). To conclude for the $\Gamma$-convergence, it is enough to show that for every $u \in L^{0} (\Omega ; \mathbb {R}^{n})$ it holds true

5.2 $$\begin{aligned} \lim _{\varepsilon \rightarrow 0} \mathcal {F}^{p}_{\varepsilon } (u, \Omega ) \le \mathcal {F}^{p} (u, \Omega )\,. \end{aligned}$$

We begin by recalling that

$$\begin{aligned} \begin{aligned} \mathcal {F}^p_{\varepsilon }(u,\Omega )&=\sup _{\mathscr {B}}\sum _{B \in \mathscr {B}}\left( \int _{\frac{\Omega -\Omega }{\varepsilon }} F_{\varepsilon ,\xi }(u,B)^p e^{-|\xi |^2} \text {d}\xi \right) ^{\frac{1}{p}}\\&=\sup _{\mathscr {B}}\sum _{B \in \mathscr {B}}\left( \int _{\frac{\Omega -\Omega }{\varepsilon }} \left( \int _{\Pi ^\xi }F_{\varepsilon }(\hat{u}_y^\xi ,B_y^\xi \cap (B-\varepsilon \xi )_y^\xi ) \text {d}y \right) ^p |\xi |^p e^{-|\xi |^2} \text {d}\xi \right) ^{\frac{1}{p}}, \end{aligned} \end{aligned}$$

where the supremum is taken over the set of finite families of disjoint open balls contained in $\Omega$.

Then, since $(B-\varepsilon \xi )^\xi _y=B^\xi _y -\varepsilon$ and since $B^\xi _y \subset \mathbb {R}$ is an interval, we combine the above equation with (5.1) and we deduce

$$\begin{aligned} \limsup _{\varepsilon \rightarrow 0}\mathcal {F}^p_{\varepsilon }(u,\Omega )&\le \limsup _{\varepsilon \rightarrow 0} \sup _{\mathscr {B}}\sum _{B \in \mathscr {B}} \frac{\pi }{2} \left( \int _{\frac{\Omega -\Omega }{\varepsilon }} \left( \int _{\Pi ^\xi } \mathcal{M}\mathcal{S}_{\frac{2}{\pi }}(\hat{u}_y^\xi ,B_y^\xi ) \text {d}y \right) ^p |\xi |^p e^{-|\xi |^2} \text {d}\xi \right) ^{\frac{1}{p}}\\&\le \sup _{\mathscr {B}} \sum _{B \in \mathscr {B}} \frac{\pi }{2} \left( \int _{\mathbb {R}^n} \left( \int _{\Pi ^\xi } \mathcal{M}\mathcal{S}_{\frac{2}{\pi }}(\hat{u}_y^\xi , B_y^\xi ) \text {d}y \right) ^p |\xi |^p e^{-|\xi |^2} \text {d}\xi \right) ^{\frac{1}{p}}. \end{aligned}$$

Thanks to Lemma 4.6 we infer (5.2). This concludes the proof of the theorem. $\square$

### Convergence of quasi-minimisers

For the purposes of this subsection, we fix two open sets $\Omega \subset \Omega ' \subset \mathbb {R}^n$. We further assume that $\partial _D \Omega$, namely, the Dirichlet part of the boundary, satisfies $\partial _D \Omega = \partial \Omega \cap \Omega '$. As it is customary in free discontinuity problems, we consider a relaxed boundary condition on $\partial _D \Omega$. To this purpose, our Dirichlet datum is given by a function $f :\partial _D \Omega \rightarrow \mathbb {R}^n$ which we identify, with a slight abuse of notation, with the trace on $\partial _{D} \Omega$ of a function $f \in H^{1}(\Omega '; \mathbb {R}^n)$. The domain of our functionals is thus denoted by $L^0_f(\Omega ';\mathbb {R}^n)$ and defined as

$$ L^0_f(\Omega ';\mathbb {R}^m):= \big \{u \in L^0(\Omega ';\mathbb {R}^n) : u= f \text { a.e. in }\Omega '\setminus \Omega \big \}. $$

In addition we define for every $\varepsilon >0$ the functionals $\mathcal {F}^{p, f}_{\varepsilon }:L^{0} (\Omega '; \mathbb {R}^{n}) \rightarrow [0, \infty ]$ as

$$ \mathcal {F}^{p,f}_{\varepsilon }(u,\Omega ') := {\left\{ \begin{array}{ll} \mathcal {F}^p_{\varepsilon }(u,\Omega ') &  \text {if } u \in L^0_f(\Omega ';\mathbb {R}^n) \\ + \infty &  \text {otherwise in } L^0(\Omega ';\mathbb {R}^n) \end{array}\right. } $$

and the limit functional $\mathcal {F}^{p,f} :L^{0} (\Omega '; \mathbb {R}^{n}) \rightarrow [0, \infty ]$ as

$$ \mathcal {F}^{p,f}(u,\Omega ') := {\left\{ \begin{array}{ll} \mathcal {F}^p(u,\Omega ') &  \text {if } u \in \text {GSBD}(\Omega ') \cap L^0_f(\Omega ';\mathbb {R}^n) \\ + \infty &  \text {otherwise in } L^0(\Omega ';\mathbb {R}^n). \end{array}\right. } $$

We further observe that the functional $\mathcal {F}^{p, f}$ takes the form

$$ \mathcal {F}^{p, f} (u,\Omega ')= \int _{\Omega '} \varphi _{p} (e (u)) \, \text {d}x + \beta _{p} \bigg (\mathcal {H}^{n-1}(J_u \cap \Omega ) + \mathcal {H}^{n-1}(J_u \cap \partial _D \Omega )\bigg ), $$

and that the term $\mathcal {H}^{n-1}(J_u \cap \partial _D \Omega )$ penalizes the part of $\partial _D \Omega$ on which the Dirichlet condition is not attained, namely, the set $\{x \in \partial _D \Omega : u^-(x) \ne f(x) \}$ where $u^-(x)$ denotes the trace with respect to the inner normal to $\partial \Omega$ of *u* at *x*.

We have the following theorem.

#### Theorem 5.2

Let $\Omega , \Omega ' \subset \mathbb {R}^n$ be two open sets, let $\partial _D \Omega = \partial \Omega \cap \Omega '$ be the Dirichlet part of the boundary, and let $f \in H^{1}(\Omega '; \mathbb {R}^n)$ be an admissible Dirichlet datum as above. Assume that $\{u_\varepsilon \}_{\varepsilon >0}$ is a family of quasi-minimisers for $\{\mathcal {F}^{p,f}_\varepsilon \}_{\varepsilon >0}$, namely,

5.3 $$\begin{aligned} \lim _{\varepsilon \rightarrow 0} \bigg ( \mathcal {F}_\varepsilon ^{p,f}(u_\varepsilon ,\Omega ')- \inf _{w \in L^0(\Omega ';\mathbb {R}^n)} \mathcal {F}_\varepsilon ^{p,f}(w,\Omega ') \bigg ) = 0\,. \end{aligned}$$

Then, there exist a minimizer $u \in \textrm{GSBD} (\Omega ') \cap L^{0}_{f} (\Omega '; \mathbb {R}^{n})$ of $\mathcal {F}^{p, f}$ and a subsequence $\varepsilon _{k} \rightarrow 0$ as $k \rightarrow \infty$ such that $u_{\varepsilon _k}\rightarrow u$ almost everywhere in $\Omega '$.

#### Proof

By (5.2) we have that $\sup _{\varepsilon >0}\mathcal {F}^{p,f}_\varepsilon (f,\Omega ') < \infty$. Hence, condition (5.3) yields $\sup _{\varepsilon >0}\mathcal {F}^{p,f}_\varepsilon (u_\varepsilon ,\Omega ') < \infty$. We are thus in position to apply Theorem 1.1 to infer the existence of a subsequence $\varepsilon _k \rightarrow 0$ as $k \rightarrow \infty$ and a finite perimeter set $A \subset \Omega '$ such that $A = \{ x \in \Omega ': \, |u_{\varepsilon _{k}}| \rightarrow \infty \text { as }k \rightarrow \infty \}$, $u_{\varepsilon _k} \rightarrow u$ pointwise a.e. in $\Omega ' \setminus A$ for $u \in \text {GSBD} (\Omega ')$ with $u=0$ in *A*. In addition, (1.7) is fulfilled. Since $u_\varepsilon = f$ a.e. in $\Omega ' \setminus \Omega$ for every $\varepsilon >0$, we deduce $A \subset \Omega$.

It remains to prove that *u* is actually a minimiser of $\mathcal {F}^{p,f}(\cdot ,\Omega ')$. We observe that we cannot immediately infer that *u* is a minimiser from the $\Gamma$-convergence given by Theorem 1.2, since the sequence $u_{\varepsilon _k}$ does not convergence in measure to *u* in the whole of $\Omega '$. Nevertheless, the proof can be achieved with the very same argument as in the standard case. Indeed, take $w \in L^{0} (\Omega '; \mathbb {R}^{n})$. Since we want to prove that $\mathcal {F}^{p,f}(w,\Omega ') \ge \mathcal {F}^{p,f}(u,\Omega ')$, we assume with no loss of generality that $w \in \text {GSBD}(\Omega ') \cap L^{0}_{f} (\Omega '; \mathbb {R}^{n})$. By virtue of (1.7), (5.2), and (5.3), we estimate

$$\begin{aligned} \mathcal {F}^{p,f}(w,\Omega ')&=\lim _{k \rightarrow \infty } \mathcal {F}^{p,f}_{\varepsilon _k}(w,\Omega ') \ge \limsup _{k \rightarrow \infty } \inf _{ v \in L^0(\Omega ';\mathbb {R}^m)} \mathcal {F}_\varepsilon ^{p,f}( v,\Omega ') \\&= \limsup _{k \rightarrow \infty }\mathcal {F}_\varepsilon ^{p,f}(u_{\varepsilon _k},\Omega ') \ge \int _{\Omega '} \varphi _{p} (e(u)) \, \text {d}x + \beta _{p} \mathcal {H}^{n-1}(J_u \cup \partial ^* A) \\&\ge \int _{\Omega '} \varphi _{p} (e(u)) \, \text {d}x + \beta _{p} \mathcal {H}^{n-1}(J_u)\\&= \int _{\Omega '} \varphi _{p} ( e(u)) \, \text {d}x + \beta _{p} \bigg (\mathcal {H}^{n-1}(J_u \cap \Omega ) + \mathcal {H}^{n-1}(J_u \cap \partial _D \Omega )\bigg ) = \mathcal {F}^{p,f}(u,\Omega '). \end{aligned}$$

Thanks to the arbitrariness of *w* we deduce that $\partial ^{*}A \subset J_{u}$ and that *u* is a minimizer of $\mathcal {F}^{p, f}$. $\square$

## Data Availability

The manuscript contains no associated data.

## Appendix A.

### Proof of Proposition 3.16

For every measurable set $B \subset A$ we can consider the linear map $i_B :L^0(A) \rightarrow L^0(B)$ defined as . Clearly, being $V \subset L^0(A)$, the map $i_B$ is a well defined linear map between the vector spaces *V* and *V*(*B*), where we have set . We claim that there exists $\varepsilon _c >0$ such that for every measurable set $K \subset A$ with $\mathcal {L}^n(A \setminus K) \le \varepsilon _c$ we have that the linear map $i_K :V \rightarrow V(K)$ is injective. Indeed, fix a basis $\{v_1,\dotsc ,v_k\}$ for *V* and assume by contradiction that there exists $\varepsilon _j \searrow 0$ and measurable sets $K_j \subset A$ with $\mathcal {L}^n(A \setminus K_j) \le \varepsilon _j$ such that there exists real coefficients, not all identically zero, $\{\alpha ^j_1,\dotsc ,\alpha ^j_k\}$, satisfying

A.1

By renormalization, with no loss of generality, we may assume that for every $i=1,\dotsc ,k$ and every $j=1,2,\dotsc$ it holds true $|\alpha ^j_i| \le 1$ and there exists at least one $i(j)=1,\dotsc ,k$ such that $|\alpha ^j_{i(j)}|=1$. Up to pass to a not relabelled subsequence in *j*, we may suppose that the index *i*(*j*) does not depend on *j*, and that $\alpha ^j_i \rightarrow \alpha _i$ for every $i=1,\dotsc ,n$, for some real coefficients $\{\alpha _1,\dotsc ,\alpha _k\}$ which are not all identically zero. Therefore, since $K_j \nearrow A$ in measure we deduce that $\sum _{i=1}^k \alpha ^j_i i_{K_j}(v_i)$ (extended to zero out of $K_j$) converges to $\sum _{i=1}^k \alpha _i v_i$ pointwise a.e. in *A* as $j \rightarrow \infty$. From (A.1) we immediately deduce that $\sum _{i=1}^k \alpha _i v_i=0$ in $L^0(A)$ which gives a contradiction. The claim is thus proved.

Now assume that $\{w_j\}_{j \in \mathbb {N}} \subset V$ is a Cauchy sequence. Since $L^0(A)$ is complete we have $\textrm{d}(w_j,w) \rightarrow 0$ as $j \rightarrow \infty$ for some $w \in L^0(A)$. We want to prove that $w \in V$. Up to passing to a subsequence in *j*, the $\textrm{d}$-convergence implies $w_j \rightarrow w$ pointwise a.e. in *A* as $j \rightarrow \infty$. Therefore, for every $0<\varepsilon \le \varepsilon _c$, we apply Egorov’s Theorem to find a measurable set $K \subset A$ such that $\mathcal {L}^n(A \setminus K) \le \varepsilon$ and $w_j \rightarrow w$ uniformly on *K* as $j \rightarrow \infty$. But this means that the sequence $\{i_K(w_j)\}_{j\in \mathbb {N}}$ is a sequence belonging to the finite-dimensional vector space *V*(*K*) and converging with respect to the $L^1$-norm. Hence, we find real coefficients $\{\alpha _1^K, \dotsc ,\alpha _k^K\}$ such that $i_K(w)= \sum _{i=1}^k \alpha _i^K i_K(v_i)$, where $\{v_1,\dotsc , v_k\}$ is a basis of *V*. In order to conclude we need to show that, given a sequence of measurable sets $K_j \nearrow A$ the coefficients $\{\alpha _1^{K_j}, \dotsc ,\alpha _k^{K_j}\}$ are definitely constant as $j \rightarrow \infty$. So assume by contradiction that for every $j\ge 1$ we find $j< j_1 < j_2$ such that $\{\alpha ^{K_{j_1}}_1, \dotsc , \alpha ^{K_{j_1}}_k\} \ne \{\alpha ^{K_{j_2}}_1, \dotsc , \alpha ^{K_{j_2}}_k\}$. Now choose $j_1$ so large such that $\mathcal {L}^n(A \setminus (K_{j_1} \cap K_{j_2})) \le \varepsilon _c$. By our assumption on the non-coincidence between the $K_{j_1}$-coefficients and the $K_{j_2}$-coefficients, we deduce that the linear combination $\sum _{i=1}^k (\alpha ^{K_{j_1}}_i -\alpha ^{K_{j_2}}_i) i_{K_{j_1} \cap K_{j_2}}(v_i)$ is identically null in $L^0(K_{j_1} \cap K_{j_2})$ while its coefficients are not all identically equal to zero. But this gives a contradiction with the fact that, thanks to our previous claim, the linear map $i_{K_{j_1} \cap K_{j_2}} :V \rightarrow V(K_{j_1} \cap K_{j_2}) \subset L^0(K_{j_1} \cap K_{j_2})$ is injective. $\square$
